# Structural Evolution and Thermal Reactivity of Kerke Coal: Molecular Model Construction and Multi-Technique Characterization

**DOI:** 10.3390/molecules31203755

**Published:** 2026-10-19

**Authors:** Xiaohong Gao, Qiang Zeng

**Affiliations:** 1 Center for Underground Coal Fire, College of Ecology and Environment, Xinjiang University, Urumqi 830017, China; 2 Key Laboratory of Oasis Ecology of Ministry of Education, Xinjiang University, Urumqi 830017, China

**Keywords:** coal molecular structure, heat treatment, structural evolution, average molecular model, thermal reaction characteristics

## Abstract

To clarify the structural evolution and thermal reactivity of Yili Kerke coal, raw and heat-treated samples were analyzed. Characterization combined proximate and ultimate analyses, TG, FTIR, XPS, ^13^C-NMR, and HRTEM. A molecular model with the formula C_179_H_132_N_2_O_13_ was constructed from multiscale structural parameters to represent the average chemical structure of Kerke raw coal. After classical molecular mechanics (MM) and molecular dynamics (MD) optimization, the lowest-energy configuration sampled had a total energy of 661.38 kcal mol^−1^. The periodic aggregate model had a density of 1.31 g cm^−3^. The ignition and burnout temperatures of the coal samples generally increased after thermal treatment. The microstructural evolution exhibited distinct stages. At low temperatures, aromatic carbon dominated, with adjustments in functional group and aromatic fringe distributions. At medium temperatures, oxygen-containing components and aromatic carbon types were redistributed, and the proportion of short fringes increased. At high temperatures, aromatic structures became more concentrated, aliphatic carbon decreased, and the proportion of larger aromatic fringes increased overall. Macroscopic thermal analysis and microstructural characterization jointly reveal that molecular structural evolution is consistent with thermal reaction characteristics at different stages. These findings provided a molecular-scale basis for understanding the oxidative thermal behavior of Kerke coal.

## 1. Introduction

Coal is an important fossil fuel in the global energy mix, but spontaneous combustion during its mining, storage, and transportation can lead to coal fires that cause resource losses and pollutant emissions [1,2]. Low-temperature coal oxidation involves exothermic reactions and the transformation of reactive functional groups [3,4]. Studies of coals from different mining areas and coal seams have identified differences in functional-group composition and microcrystalline structure, alongside variations in oxidation, pyrolysis, and combustion behavior [5,6,7]. Therefore, a systematic understanding of the evolution of coal’s microscopic molecular structure during thermal processing and its relationship with macroscopic thermal reaction characteristics is a crucial foundation for deepening our understanding of the laws governing coal’s thermal transformation and promoting the safe and efficient utilization of coal.

Coal exhibits structural characteristics such as complexity, heterogeneity, and amorphousness; molecular models of coal serve as important tools for characterizing its average chemical structure and conducting studies at the molecular scale. With the advancement of coal structure research, representative structural models including the Fuchs [8], Wiser [9], Solomo [10], Given [11] and the Shinn models [12] have been successively proposed, laying the foundation for understanding the basic structural units of coal and how they are connected. In recent years, researchers both domestically and internationally have primarily employed multiple characterization techniques to obtain structural parameters of coal [13]. Elemental and industrial analyses provide a macroscopic foundation, while techniques such as ^13^C-NMR, FTIR, XPS, and HRTEM are used to analyze molecular and microscopic spatial structures, respectively [14,15,16]. Li et al. used fast Fourier transform (FFT) filtering and inverse fast Fourier transform (IFFT) processing to extract aromatic fringes from HRTEM images and quantify their length distributions [16]. Chen et al. used image filtering, binarization, and skeletonization to quantify the length and curvature of aromatic fringes in medium-rank coals. They found that short and intermediate-length fringes predominated [17]. Based on ^13^C-NMR, FTIR, and XPS results, Qian et al. constructed a molecular structural model of Zhundong subbituminous coal; they employed molecular mechanics and molecular dynamics to obtain lower-energy conformations and evaluated the model’s validity by comparing simulated spectra with experimentally measured ^13^C-NMR and FTIR spectra [18]. Shi et al. assessed a molecular model of unburned carbon from coal gasification fine slag by comparing its calculated infrared spectrum with the measured FTIR spectrum [19]. Yuan et al. constructed a coal aggregate model using the Amorphous Cell (AC) module and performed geometry optimization and simulated annealing with the Dreiding force field. NPT simulations yielded a density of 1.36 g cm^−3^, differing from the measured value by only 0.02 g cm^−3^ [20].

The macroscopic thermal reaction characteristics [20] and microstructural responses of coal under different heating conditions have also attracted widespread attention. Guo et al. divided the secondary oxidation process of pre-oxidized coal into stages of moisture evaporation, oxygen adsorption-induced weight gain, and combustion. They found that the characteristic temperatures in the low-temperature stage responded more significantly to pre-oxidation conditions, whereas the characteristic temperatures in the high-temperature combustion stage showed smaller changes; the apparent activation energies in different stages exhibited distinct trends [21]. Jia et al. found that both the pre-oxidation temperature and air flow rate influence the characteristic temperatures and apparent activation energies of coal samples in TG/DTG analyses, and that different reaction stages respond inconsistently to pretreatment conditions [22]. Zang et al. reported heating-rate-dependent characteristic temperatures and stage-dependent apparent activation energies using TG–DTG–DSC measurements and Coats–Redfern analysis [23]. In terms of microstructure, a study combining DSC and in situ FTIR showed that the functional groups most closely associated with heat release differed among reaction stages during coal oxidation and combustion [24]. Chai Shuangqi et al. conducted multiscale spectroscopic characterization on coal samples treated at temperatures ranging from 20 to 800 °C and found that the elemental speciation, functional group composition, and parameters of aliphatic and aromatic structures all underwent stage-specific changes with heat treatment temperature [25]. Through HRTEM observations, Li and Zeng found that during heat treatment, the length, curvature, orderliness, interlayer spacing, and stacking height of the coal samples’ aromatic layers changed with temperature; after treatment at 300 °C, some aromatic structural parameters showed significant differences from those of raw coal [26]. These studies revealed coal’s temperature response from the perspectives of thermal analysis, functional groups, carbon structure, and aromatic layers, respectively. These studies provide a basis for understanding the structural evolution and thermal responses of coal. The Kerke coal examined here has a higher carbon content and a lower oxygen content on a dry, ash-free basis than the ranges reported for Zhundong coal [27]. Against this compositional background, further clarification is needed of how the carbon framework, oxygen-containing functional groups, and aromatic-fringe length distribution of Kerke coal evolve during heat treatment and how these changes correspond to thermal responses at different reaction stages. Overall, structural changes in temperature sequences and macroscopic thermal reaction parameters in existing studies still largely serve as relatively independent analytical tasks; even when the relationship between the two is addressed, it often focuses on a single structural scale or a limited number of thermal reaction parameters, and the systematic connection between them still needs to be further strengthened.

This study investigated the structural evolution and thermal responses of coal from the Yili Kerke Mine. An average molecular model of Kerke raw coal was constructed using elemental and spectroscopic data as constraints. The molecular conformation was optimized using MM and MD, and a periodic aggregate model was constructed to describe molecular packing. Raw coal and samples heat-treated at 50–500 °C were examined using TG/DTG and multiscale structural characterization. This study analyzed changes in characteristic temperatures and apparent activation energies, and examined the temperature responses of functional group composition, carbon backbone structure, and aromatic fringe length distributions, thereby investigating the correspondence between molecular structural evolution and macroscopic thermal reaction characteristics at different temperature stages.

## 2. Results and Discussion

### 2.1. Composition and Molecular Structural Characteristics of Raw Coal

#### 2.1.1. Composition Analysis

The results of the industrial and elemental analyses are shown in Table 1. The elemental composition and atomic ratios can provide a basis for constructing molecular models of raw coal.

#### 2.1.2. ^13^C-NMR Test Results

**Figure 1:**
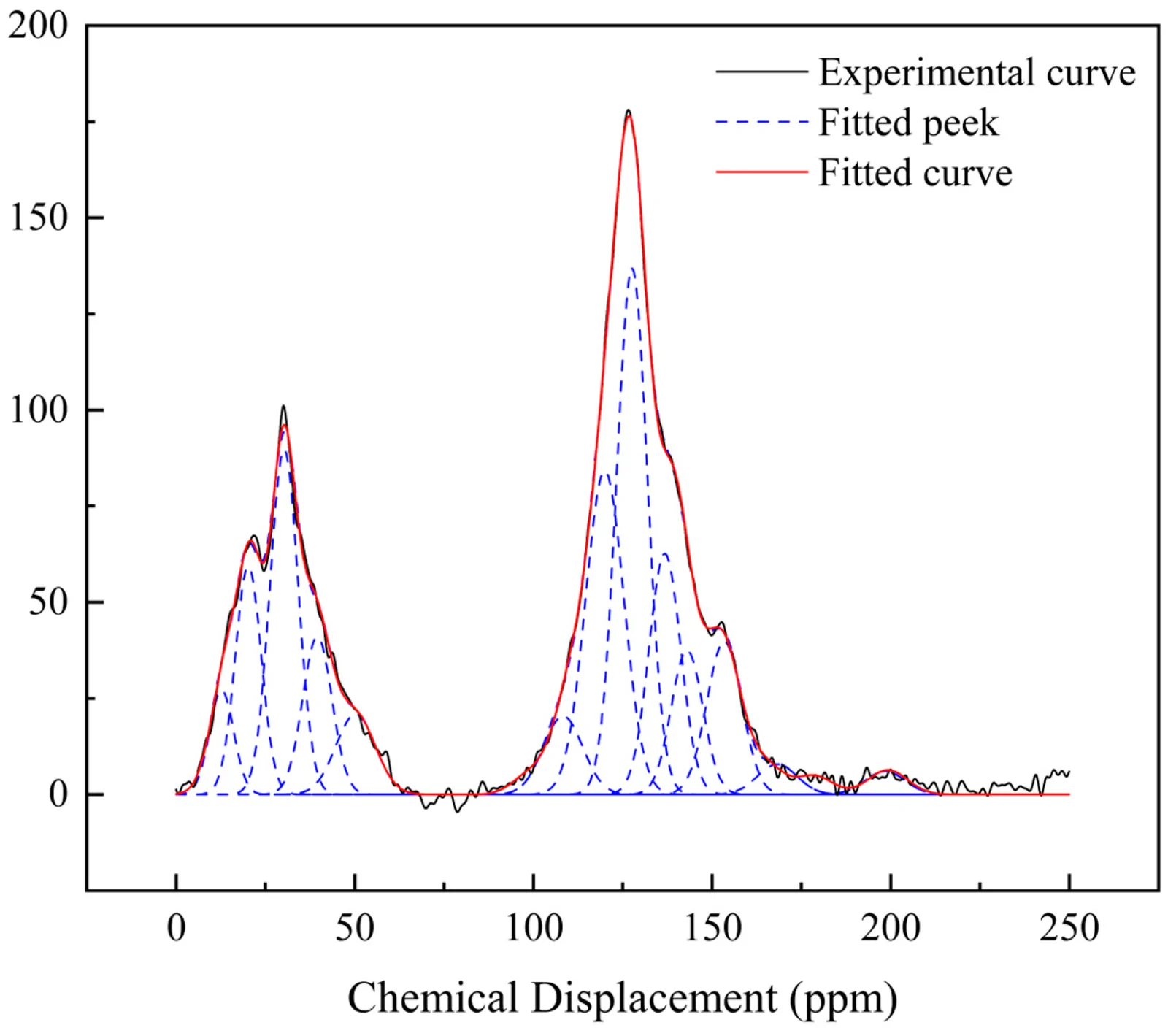
^13^C-NMR spectral fitting results.

Solid-state ^13^C NMR can quantitatively characterize the relative distribution of different carbon structures in coal, providing a basis for identifying the composition of the carbon skeleton in raw coal and the degree of aromatic condensation [28]. Peak fitting of the ^13^C-NMR spectra of raw coal from the Kerke Mine was performed using Origin 2024, yielding a coefficient of determination (R^2^) of 0.997. The fitting results are shown in Figure 1. The chemical shifts, peak assignments, and relative peak areas of the characteristic peaks for each carbon structure are listed in Table 2, and the carbon structure parameters of the raw coal are presented in Table 3.

The spectrum is dominated by the aliphatic carbon signal band (0–60 ppm) and the aromatic carbon signal band (100–165 ppm), with the aromatic carbon signal area significantly higher than that of the aliphatic carbon, indicating that aromatic carbon constitutes the main component of the raw coal’s carbon skeleton. Table 2 shows that aromatic carbon (f_a_) and aliphatic carbon (f_al_) account for 66.34% and 33.66%, respectively, indicating that the raw coal’s carbon skeleton is predominantly aromatic. Aromatic bridgehead carbon (f_a_^B^) accounts for 9.91%, while aliphatic carbon consists primarily of methylene and methyl carbon. The ratio of aromatic bridgehead carbon to surrounding aromatic carbon, X_BP_, reflects the degree of aromatic ring condensation; it is calculated as $X_{\mathrm{BP}}=f_{a}^{B}/\left(f_{a}^{H}+f_{a}^{P}{+f}_{a}^{S}\right)$. In this study, X_BP_ was 0.185, indicating a low proportion of bridging aromatic carbon and a limited degree of condensation in the raw coal’s aromatic structure. These parameters collectively constitute the carbon structural constraints for the subsequent construction of the raw coal molecular model.

#### 2.1.3. FTIR Test Results

**Figure 2:**
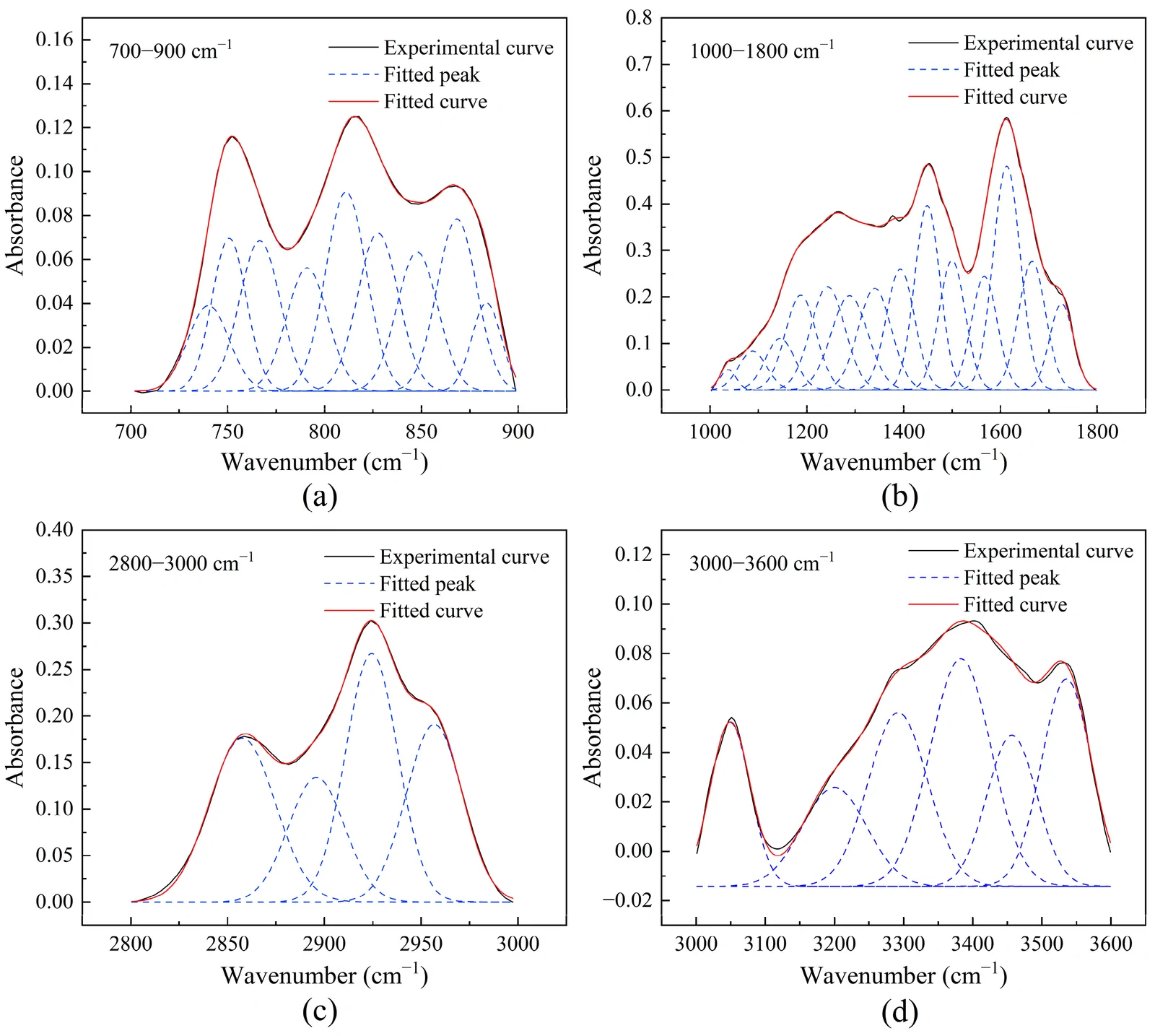
FTIR fitting spectra of raw coal: (**a**) 700–900 cm^−1^; (**b**) 1000–1800 cm^−1^; (**c**) 2800–3000 cm^−1^; (**d**) 3000–3600 cm^−1^.

FTIR can be used to characterize the types and relative distributions of functional groups in coal, providing a basis for extracting parameters for the molecular model of raw coal. As shown in Figure 2, the FTIR spectra of coal samples can be divided into the aromatic ring substitution region (700–900 cm^−1^), the oxygen-containing functional group region (1000–1800 cm^−1^), the aliphatic region (2800–3000 cm^−1^), and the hydroxyl region (3000–3600 cm^−1^). Gaussian functions were used to perform peak fitting on each spectral region; the coefficients of determination (R^2^) for the fits were all greater than 0.995. The proportions of stretch vibration areas for each functional group were calculated and were primarily used to incorporate oxygen-containing functional groups into the molecular model [29]. Peak assignments and relative peak areas are shown in Table 4.

In the aromatic ring substitution region (700–900 cm^−1^), tetrasubstitution accounted for 40.89%, indicating that tetrasubstitution is the predominant form of benzene ring substitution; in the 1000–1800 cm^−1^ region corresponding to oxygen-containing functional groups, the combined relative area of the C-O vibrational peaks was 30.45%, higher than the 13.08% for the C=O vibrational peaks, indicating that oxygen atoms in raw coal are primarily present in the form of C-O bonds, while the proportion of carbonyl structures is relatively low; in the aliphatic region (2800–3000 cm^−1^), CH_3_ stretching vibrations accounted for 50.97%, while CH_2_ asymmetric stretching vibration accounted for 31.81%, indicating that methyl and methylene groups are the predominant groups in the coal; in the hydroxyl region (3000–3600 cm^−1^), ether hydrogen bonds accounted for the largest proportion at 44.57%, indicating strong associative interactions between hydroxyl groups and ether oxygen atoms.

#### 2.1.4. XPS Test Results

**Figure 3:**
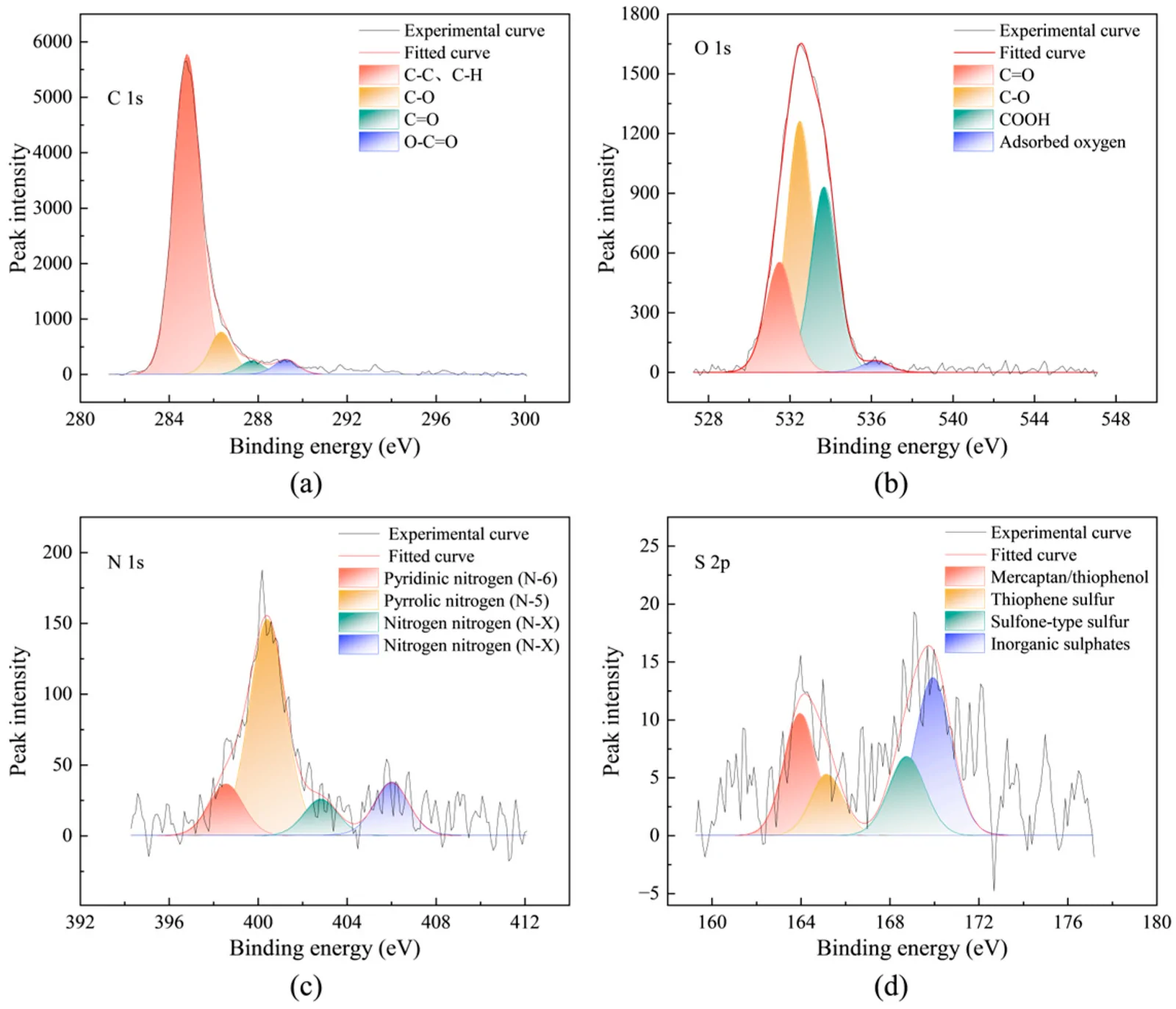
XPS spectra fitting for raw coal: (**a**) C 1s spectrum; (**b**) O 1s spectrum; (**c**) N 1s spectrum; (**d**) S 2p spectrum.

XPS analysis was conducted to determine the distribution and chemical environment of the elements C, O, N, and S on the surface of the Kerke raw coal. Peak fitting was performed using Avantage 6.9.0, with the binding energy calibrated using the C 1s main peak at 284.8 eV. The fitting results and relative peak areas are shown in Figure 3 and Table 5.

The analysis results show that, in the C 1s spectrum, the C-C/C-H components account for 83.62%, indicating that the carbon framework on the surface of raw coal consists primarily of non-oxygen-containing carbon structures. In the O 1s spectrum, the C-O component accounted for 44.75%, representing the primary form of organic oxygen; the COO-/COOH and C=O components accounted for 33.02% and 20.22%, respectively, while adsorbed oxygen accounted for only 2.00%. These results indicate that organic oxygen on the raw coal surface primarily exists in the form of C-O bonds, while also retaining a certain proportion of carboxyl and carbonyl groups. In the N 1s spectrum, nitrogen exists in three forms: pyrrolic nitrogen (N-5), pyridinic nitrogen (N-6), and oxidized nitrogen (N-X), with proportions of 61.78%, 13.95%, and 24.27%, respectively; quaternary nitrogen (N-Q) was not detected. Based on the ^13^C-NMR results, the aromatic carbon fraction (f_a_) was 66.34%. The sample is classified as a medium-rank coal with limited aromatic condensation; the absence of N-Q is consistent with the evolution patterns of nitrogen forms in medium-rank coals [30]. Furthermore, the sample surface is rich in oxygen-containing functional groups; N-6 and N-5 are easily oxidized to form oxidized nitrogen, resulting in a relatively high N-X content [31]. In the S 2p spectrum, inorganic sulfates predominated (39.26%), followed by thiols and thiophenols (27.37%), sulfone sulfur (19.68%), and thiophene-type sulfur (13.69%).

#### 2.1.5. Aromatic Fringe Structure

**Figure 4:**
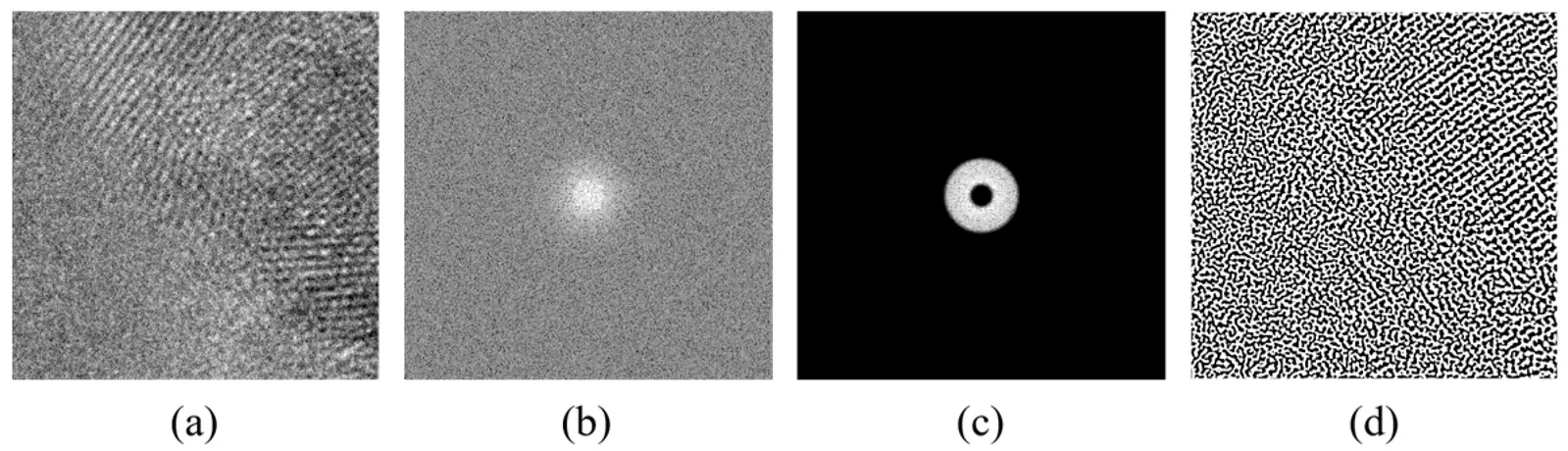
Key images in HRTEM image processing: (**a**) original image; (**b**) frequency-domain image; (**c**) annular-filtered image; (**d**) binarized image.

During HRTEM testing, factors such as voltage fluctuations, energy loss caused by electron scattering from the sample, and thermal drift of the sample result in significant noise in the raw images, making it difficult to accurately identify graphite aromatic fringes. A series of image processing steps is therefore required to obtain accurate information on the aromatic ring structure. First, Digital Micrograph is used to adjust the contrast of the raw HRTEM images, thereby reducing the impact of noise on the identification of aromatic fringes. Based on this, a two-dimensional FFT is performed to obtain a frequency-domain image; the annular diffraction features observed in the frequency domain correspond to the statistical distribution characteristics of graphite-like aromatic layers in coal. The frequency-domain image was subjected to annular filtering to enhance aromatic lattice-fringe contrast. Subsequently, an IFFT was applied to enhance the image’s periodicity, making the distribution of aromatic fringes clearer. Python code was then used to perform Gaussian-weighted local adaptive thresholding (45 × 45-pixel window; offset = 0.035), converting the enhanced image into a binary image to separate the valid striations from the background; the binary image underwent skeletonization to remove isolated noise points. The study indicates that the molecular size of 1 × 1 aromatic layer ring is approximately 0.3 nm. Segments shorter than 0.25 nm were excluded from the fringe-length statistics, and the retained fringes were represented as one-pixel-wide skeletons. The key processed image is shown in Figure 4. Aromatic fringes identified using the above procedure were classified categories ranging from 1 × 1 to 8 × 8 for fringe length distribution analysis [32].

### 2.2. Model Development and Optimization

#### 2.2.1. Determination of Structural Parameters

- Aromatic structures

Ultimate analysis indicates that the carbon content in the Kerke coal is 81.66%; this value serves as a constraint on the elemental composition of the model. The X_BP_ of Kerke coal is 0.185, falling between the characteristic values of benzene and naphthalene rings. Combined with the ^13^C-NMR results, the model’s aromatic skeleton consists primarily of benzene and naphthalene rings, with a small amount of phenanthrene or anthracene rings; the number of each aromatic structural unit is shown in Table 6. Based on the total number of carbon atoms in the model (179), the f_a_ value is 66.34%, corresponding to approximately 119 aromatic carbon atoms. By adjusting the relative numbers of each aromatic structural unit to bring the model’s X_BP_ close to the experimental value, the degree of aromatic condensation is constrained.

- Aliphatic structure

Aliphatic carbon atoms are primarily distributed around the aromatic structure in the form of side chains, bridging chains, and naphthenes. The f_al_ value is 33.66%, corresponding to approximately 60 aliphatic carbon atoms. The proportions of f_al_^H^, f_al_^O^, and f_al_^*^ are 18.60%, 4.09%, and 10.97%, respectively. Based on the characteristic CH_3_ and CH_2_ absorption peaks in the FTIR spectrum, the aliphatic structure consists primarily of methyl and methylene groups, with a small amount of oxygen-containing aliphatic carbon retained. Based on the elemental atomic ratios, the number of hydrogen atoms in the model is set to 132.

- Heteroatomic structure

Based on the atomic ratios of the elements, the model includes 13 O atoms and 2 N atoms; since the number of S atoms is less than 1, it was not included in the model. FTIR and ^13^C-NMR analyses indicate that the raw coal contains multiple types of oxygen-containing functional groups. The FTIR band assignments and relative peak areas reported in Table 4 informed the initial allocation of hydroxyl and ether groups in the model. The O 1s results from XPS show that the contents of C-O, COO-/COOH, and C=O are 44.75%, 33.02%, and 20.22%, respectively. The XPS surface data were used as an approximate reference alongside the ^13^C-NMR results and the total oxygen atom count. Based on this, the model was configured with 2 hydroxyl groups, 2 carbonyl groups, 3 ether linkages, and 3 carboxyl groups, containing a total of 13 O atoms. The N 1s spectrum in XPS showed that the relative abundances of N-5, N-6, and N-X were 61.78%, 13.95%, and 24.27%, respectively. The molecular model contains one pyrrolic nitrogen and one pyridinic nitrogen atom. N-X was not included as a separate structural unit. The resulting molecular formula is C_179_H_132_N_2_O_13_.

#### 2.2.2. Construction and Validation of Planar Molecular Structure Models

**Figure 5:**
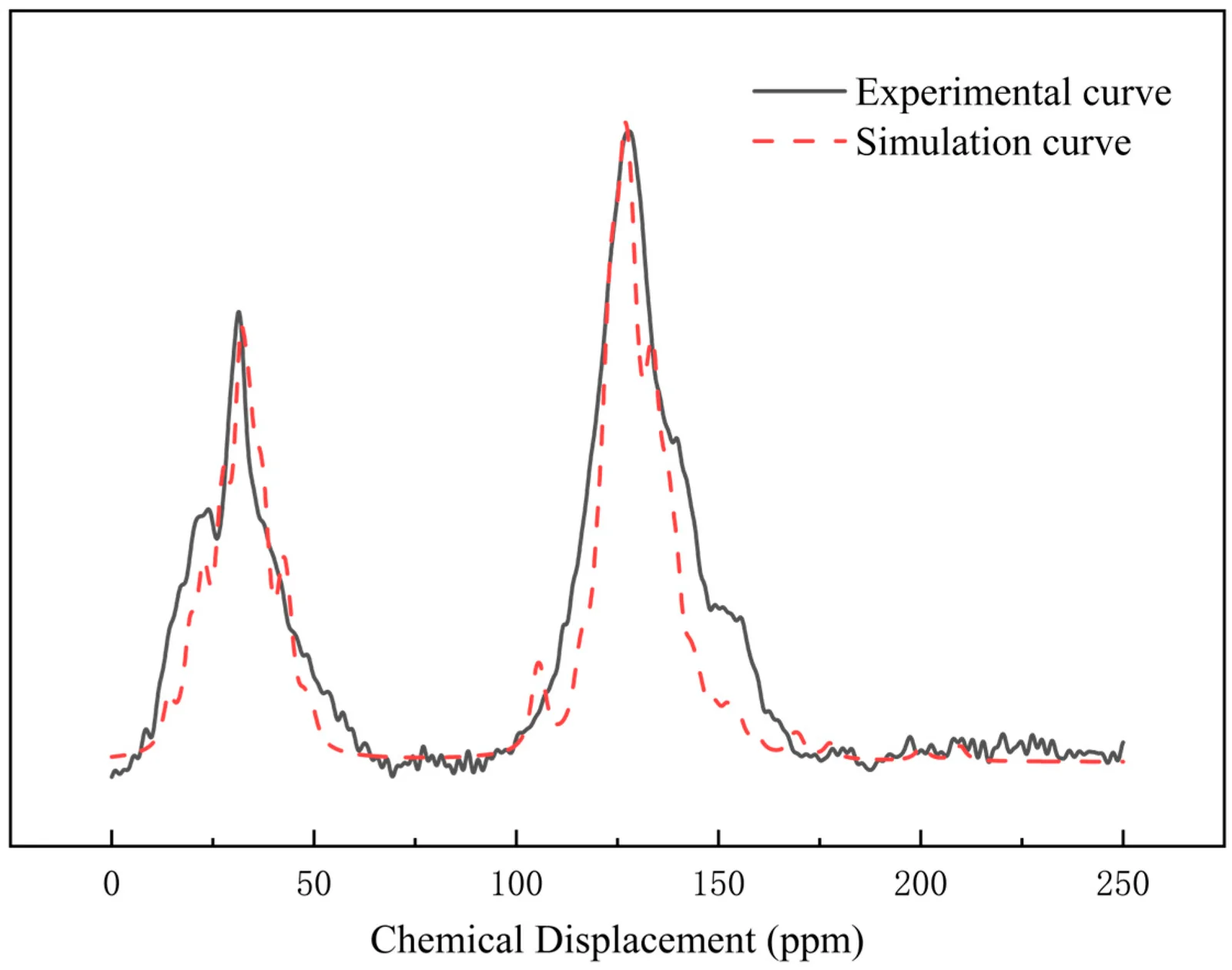
Comparison of ^13^C-NMR spectra of the Kerke coal model.

**Figure 6:**
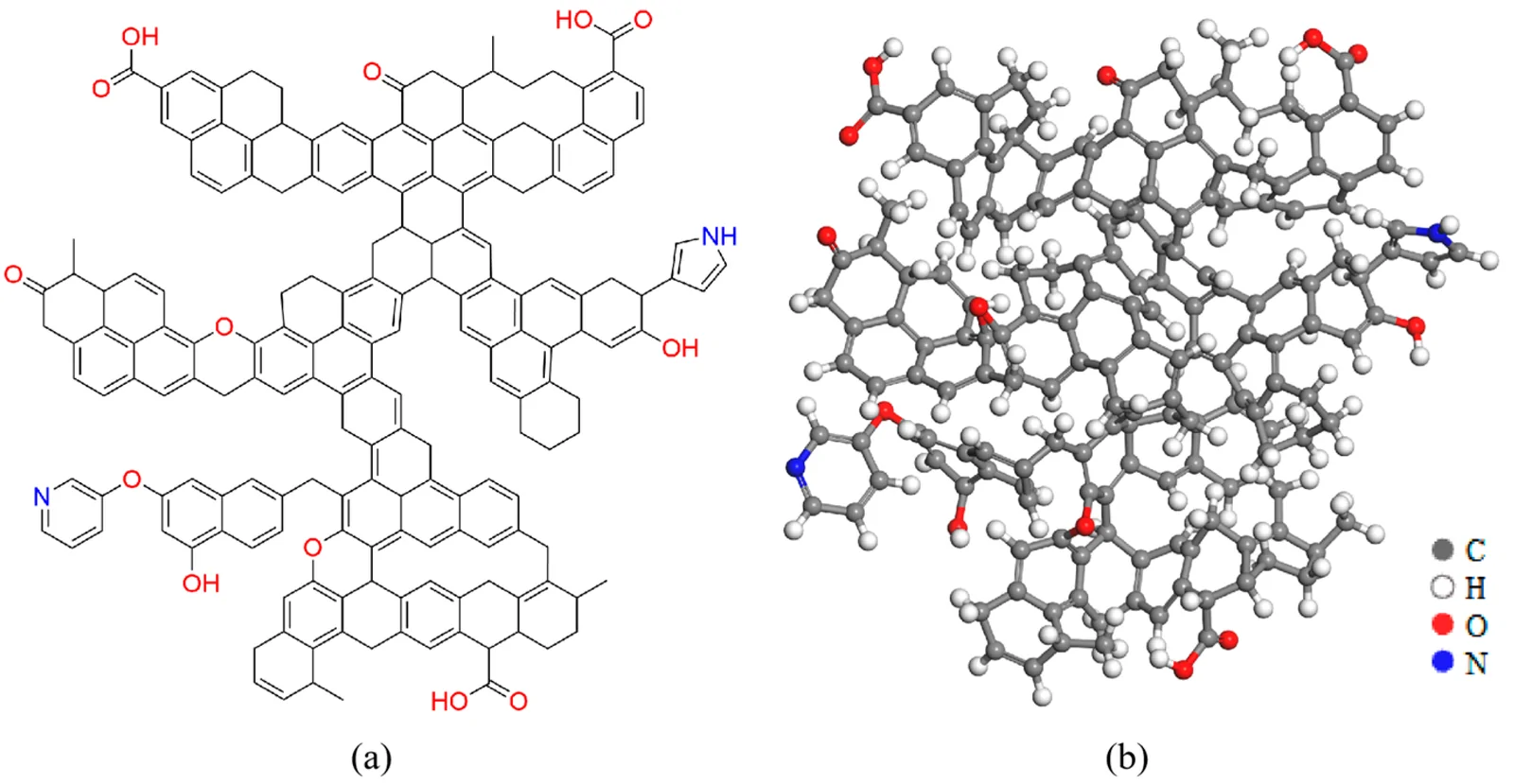
Planar structure of a coal molecule (**a**); initial 3D model (**b**).

A two-dimensional molecular structure model of Kerke raw coal was constructed using ChemDraw 23.1.1. The aromatic nucleus was built based on the aromatic structural units listed in Table 6. Combining the results from ^13^C-NMR, FTIR, and XPS, the aliphatic side chains, bridging structures, and oxygen- and nitrogen-containing functional groups were arranged around the aromatic skeleton. To verify the accuracy of the constructed molecular model, MestReNova 15 was used to predict the ^13^C chemical shifts of the two-dimensional planar structure. The bonding patterns of the various functional groups in the coal molecule were continuously adjusted until the predicted spectrum matched the experimental spectrum, as shown in Figure 5. The constructed planar structure of the raw coal molecule and the initial 3D model are shown in Figure 6, and the model construction information is presented in Table 7, which corresponds to the normalized results of the ultimate analysis. Further comparison of theoretical infrared spectra obtained from vibrational frequency calculations in Gaussian with experimental FTIR spectra would provide complementary spectroscopic evidence for assessing the functional-group characteristics represented by the model.

#### 2.2.3. Model Optimization and Energy Analysis

**Figure 7:**
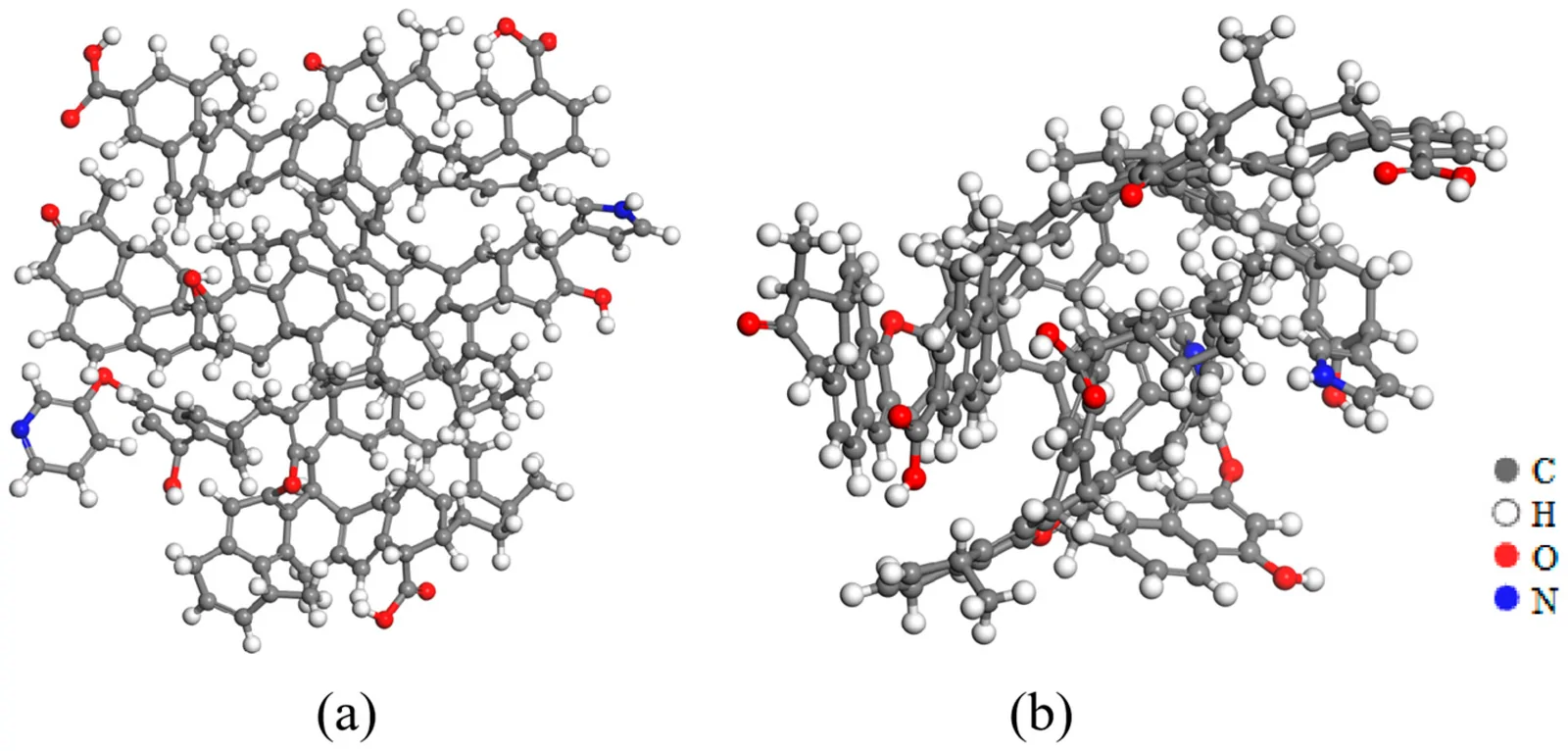
Model structure after geometric optimization (**a**) and annealing simulation (**b**).

The initial Kerke coal model was optimized in Materials Studio (2024) using MM and MD for energy minimization and structural equilibration. The configurations obtained after geometry optimization and annealing are shown in Figure 7. Both geometry optimization and simulated annealing were performed using the Universal force field and the Ultra-fine quality setting. Atomic charges were assigned using the Gasteiger method. Geometry optimization employed the Smart algorithm with a maximum of 5000 iterations. The energy and force convergence tolerances were set to 2.0 e^−5^ kcal mol^−1^ and 0.001 kcal mol^−1^ Å^−1^, respectively. Simulated annealing was performed for 20 cycles over a temperature range of 300–600 K in the NVT ensemble using the Nosé thermostat. The integration time step was 1.0 fs, and the total simulation time was 2400 ps. The energies of the model before and after optimization are shown in Table 8. After optimization, the valence electron energy and non-bonding energy decreased, the bond stretching energy decreased, and the total energy of the model was significantly reduced. Among these, the van der Waals energy is the major contributor to the total energy, and the stability of the model structure primarily depends on the van der Waals energy. The geometric optimization and annealing process effectively eliminated the local high-energy structures in the initial configuration, causing the molecular conformation to become more stable.

#### 2.2.4. Construction of Condensed-Phase Models

**Figure 8:**
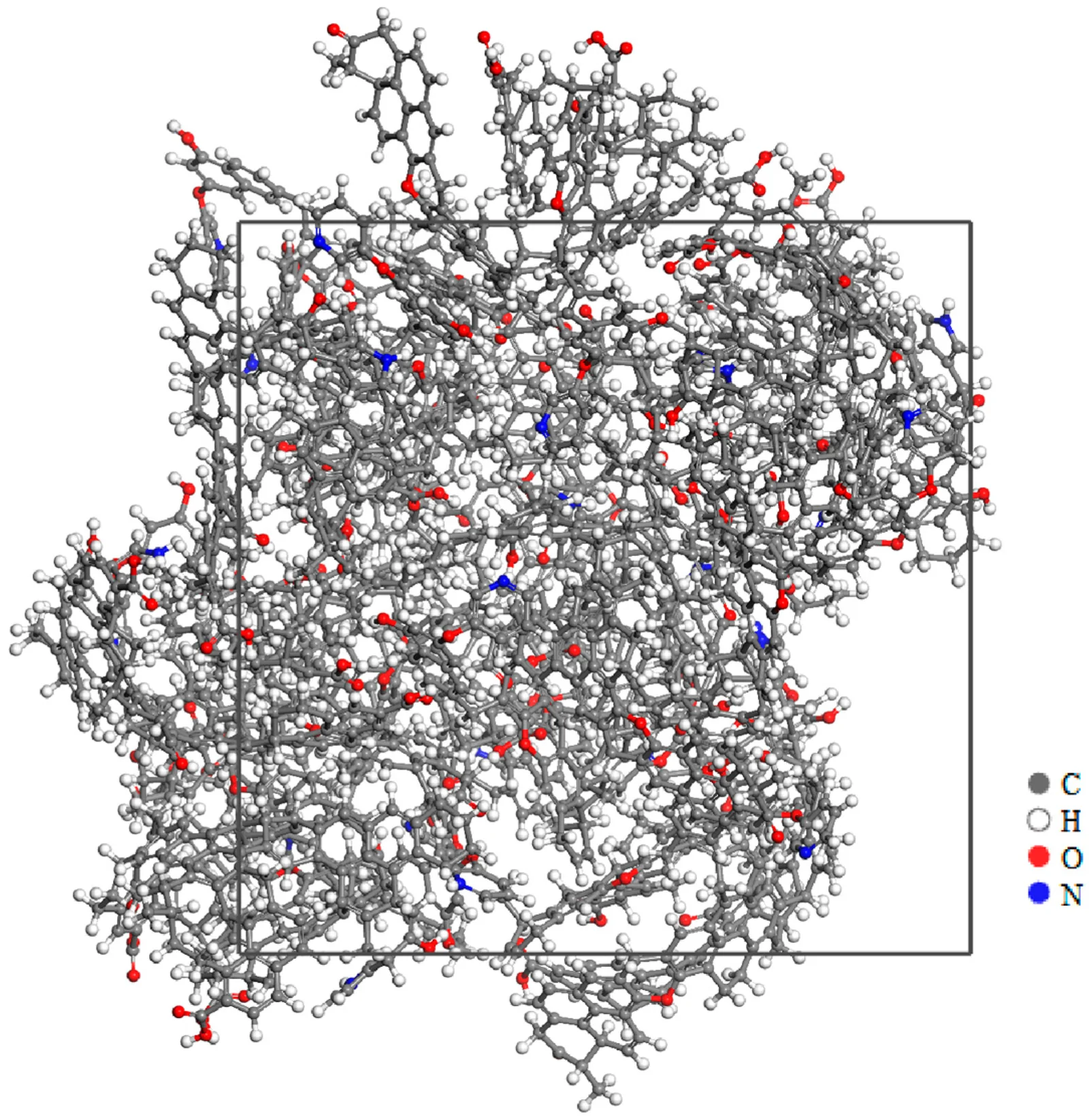
Structural model of molecular aggregates in Kerke coal.

Using the AC module in MS 2024, 12 optimized molecular structural models of Kerke coal were placed in a periodic cubic box measuring 46.5 Å × 46.5 Å × 46.5 Å (1 Å = 0.1 nm), with the initial density of the structural models set to 0.5 g/cm^3^. First, the NVE system was used to achieve low-temperature kinetic equilibrium in the initial system to prevent atomic overlap. Subsequently, the system was compacted at 1.2 GPa in the NPT ensemble using a Nosé thermostat and a Berendsen barostat for temperature and pressure control, respectively. The resulting model density was 1.31 g cm^−3^, within the density range of bituminous coal (1.15–1.509 g/cm^3^). Finally, an annealing kinetics simulation was performed to subject the system to NVT relaxation using a Nosé thermostat for temperature control. The configuration with the lowest energy was selected as the structural model of the coal molecular aggregate under periodic boundary conditions, as shown in Figure 8. The optimized aggregate model represents one possible molecular packing configuration of Kerke coal under periodic boundary conditions. Its spatial configuration was obtained through geometry optimization and structural relaxation. This model will be used in subsequent work to perform ReaxFF simulations.

### 2.3. Analysis of the Thermal Reaction Characteristics of Coal Samples

#### 2.3.1. TG-DTG and Characteristic Temperature Points

**Figure 9:**
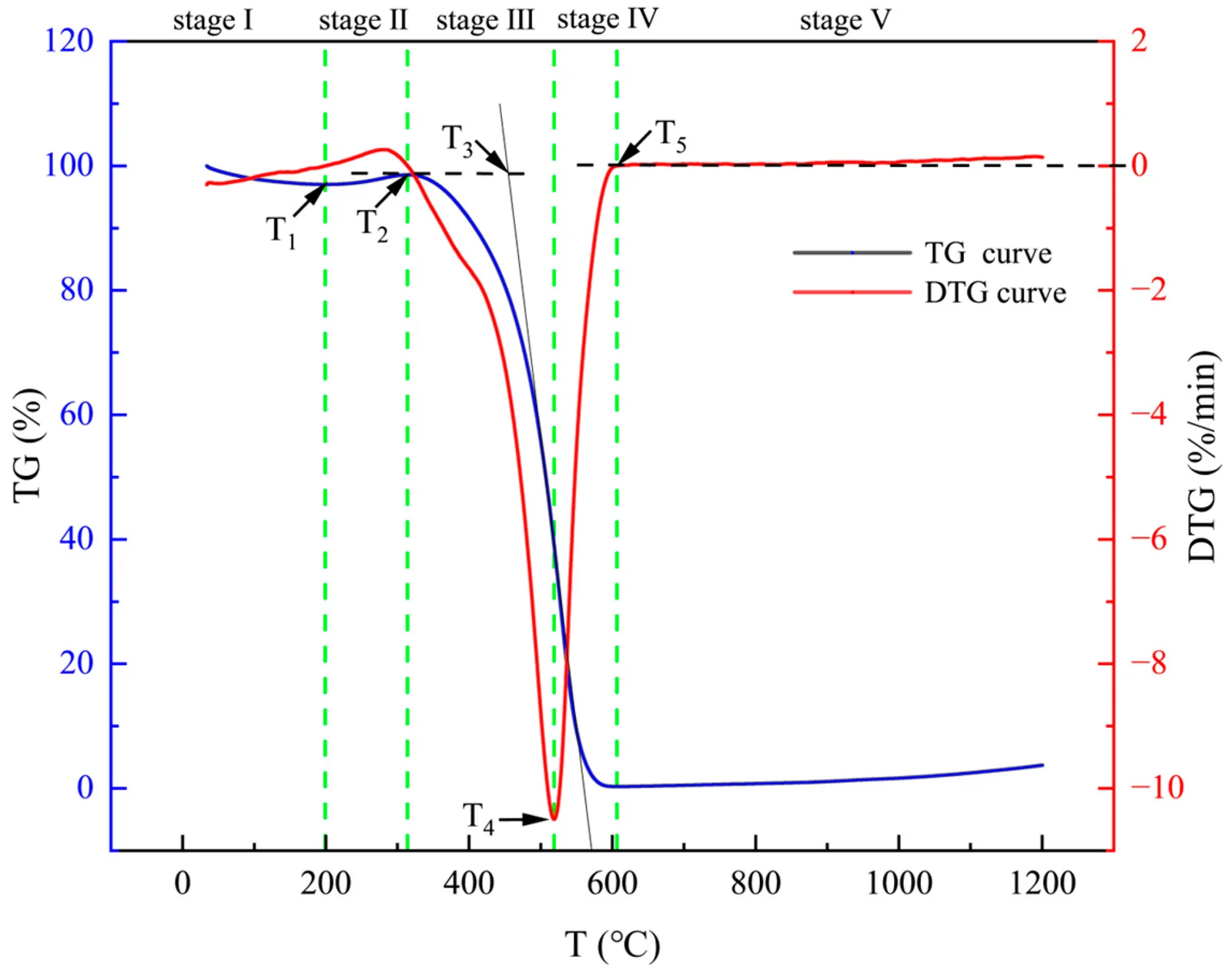
Raw coal TG-DTG curves.

Based on the characteristics of the TG-DTG curves during the coal oxidation combustion process, the following characteristic temperature points were determined: T_1_ is the inflection point temperature at which the coal sample loses water and begins to gain weight due to oxygen absorption; T_2_ is the onset temperature of pyrolysis; T_3_ is the ignition temperature; T_4_ is the temperature at which the maximum combustion rate is reached; and T_5_ is the complete burnout temperature. Based on the characteristic temperature points, the coal oxidation combustion process is divided into five stages: the moisture evaporation and gas desorption stage (Stage 1), the oxygen absorption and weight gain stage (Stage 2), the thermal decomposition stage (Stage 3), the combustion stage (Stage 4), and the complete combustion stage (Stage 5), as shown in Figure 9.

The characteristic temperature points of coal samples treated at different temperatures are shown in Table 9. T_1_ fluctuates irregularly with heat treatment temperature, generally showing a trend of first rising, then falling, reaching a minimum at 300 °C, and then rising again before falling once more. This may be due to differences in moisture content among coal samples with varying degrees of oxidation. T_2_ and T_3_ show little variation in coal samples treated at low to medium temperatures but begin to rise above 400 °C. The T_3_, T_4_, and T_5_ values of the heat-treated coal samples are all higher than those of the raw coal, with relatively minor fluctuations overall. This is primarily because, as the temperature rises, the more reactive aliphatic hydrocarbon functional groups in the coal samples gradually decrease, and the aromatic hydrocarbons are consumed, making it more difficult for the substances to react. Consequently, the reactivity of these substances decreased, resulting in only slight changes in the overall inflection point temperatures.

#### 2.3.2. Analysis of Thermal Reaction Kinetics

**Figure 10:**
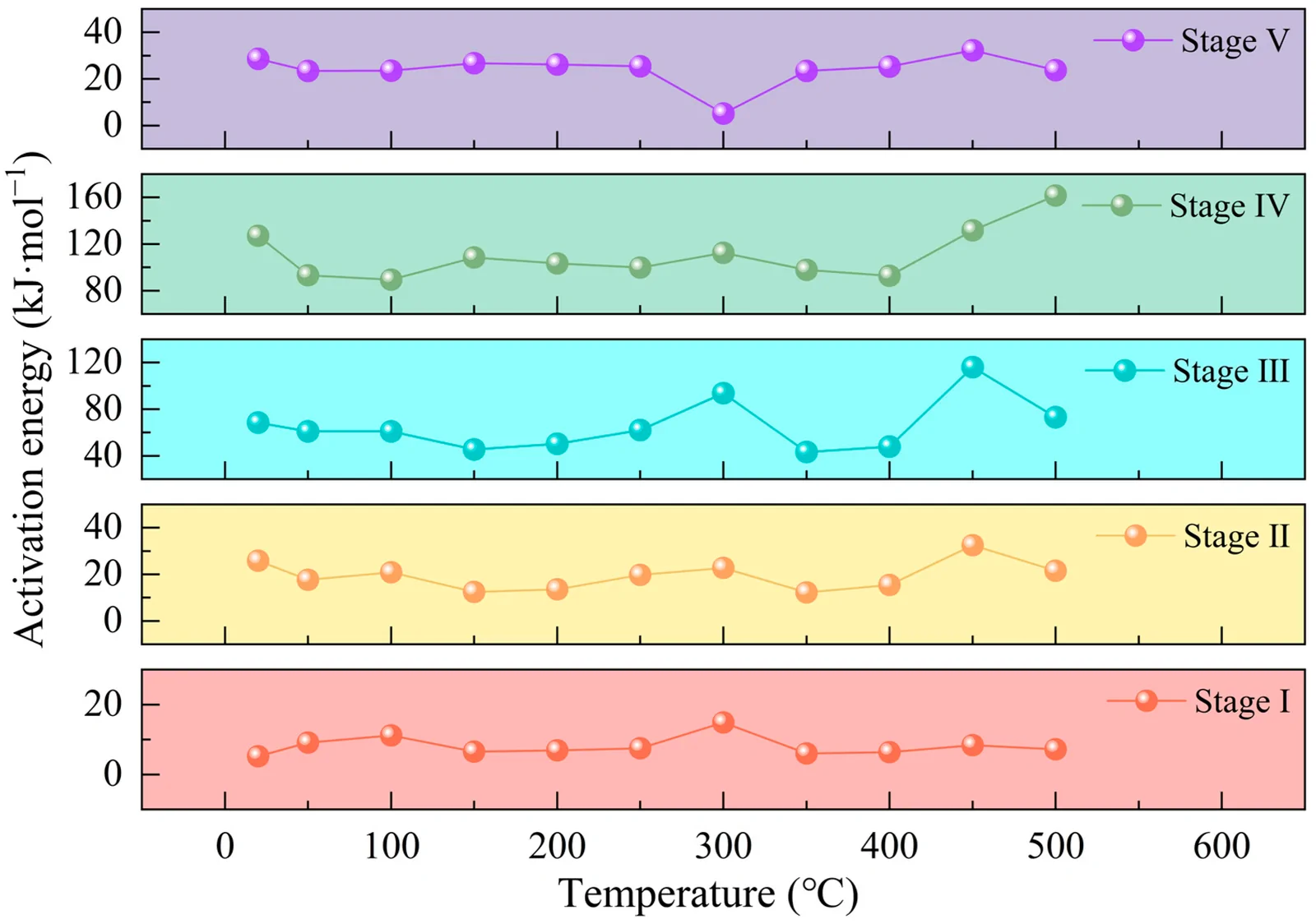
Activation energy of coal samples at different temperatures.

The apparent activation energy of the heat-treated coal samples was calculated using the Coats-Redfern equation, and the results are shown in Figure 10.

The apparent activation energy for Stage IV was consistently the highest across all coal samples and peaked at 500 °C, indicating that this stage has a higher apparent energy barrier. The activation energy for Stage III exhibited significant variation, with local decreased observed near 300 °C and 450 °C, suggesting that heat treatment had a relatively pronounced effect on the kinetic response of this stage. In contrast, the activation energies for Stages I, II, and V were generally lower and exhibited only non-monotonic fluctuations. These results indicate that heat treatment did not cause synchronous changes in the reaction ease across all reaction stages but primarily altered the apparent kinetic characteristics of Stages III and IV; the structural basis for this requires further verification through characterization of functional groups and carbon structures.

### 2.4. Temperature-Dependent Characteristics of Coal Molecular Structures

#### 2.4.1. Evolutionary Characteristics of Carbon Scaffold Structures

**Figure 11:**
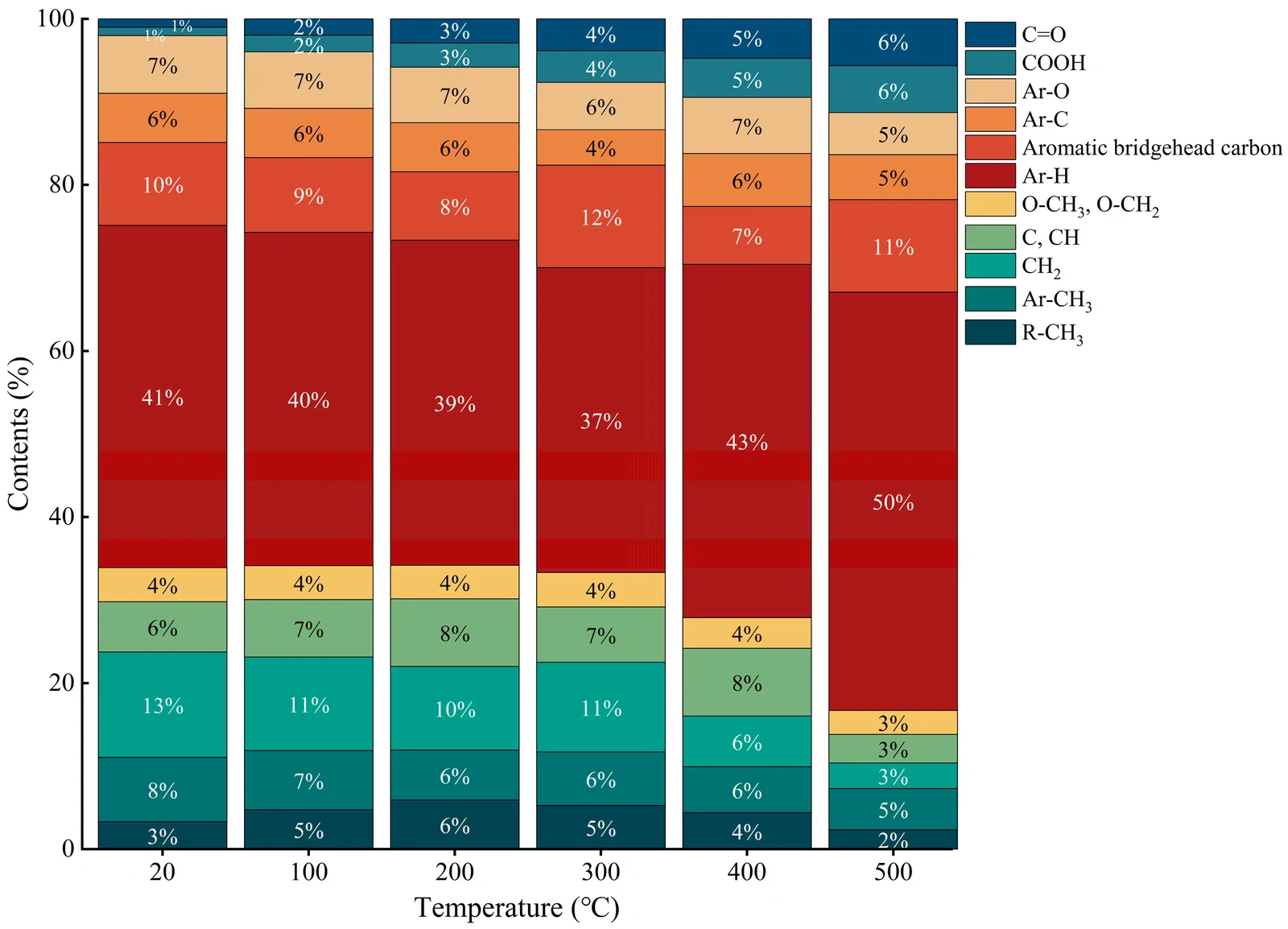
Changes in the C structure of coal samples at different temperatures.

Based on the macroscopic reaction stages and characteristic transformations during the oxidative combustion of coal, and taking into account the temperature gradients and structural parameter changes in the heat-treated samples, the ^13^C-NMR, FTIR, XPS, and HRTEM results were classified into three stages: low temperature (20–200 °C), medium temperature (200–300 °C), and high temperature (300–500 °C). ^13^C-NMR measurements were performed on coal samples heated to temperatures ranging from 20 to 500 °C to analyze the evolution of the coal’s molecular carbon framework and functional groups. The changes in the relative peak area of C structures in coal samples at different temperatures are shown in Figure 11.

During the low-temperature stage, the carbon framework remained predominantly aromatic, with Ar-H as the main carbon component. The relative peak areas of R-CH_3_, C=O, and COOH, increased, whereas those of Ar-CH_3_ and CH_2_ decreased. However, fluctuations in the content of each component do not exceed 2%, indicating that the carbon framework has not undergone significant changes, but is merely accompanied by slight thermal motion and the removal of adsorbed water.

During the medium-temperature stage, the relative peak areas of CH_2_, bridging aromatic carbon, and C=O gradually increased, while the relative peak areas of Ar-H, R-CH_3_, C, CH decreased. These changes indicate a redistribution among aromatic carbon environments, with a greater relative contribution from bridgehead sites associated with condensed aromatic units. The carbonyl increase further suggests concurrent oxidative modification of the coal structure.

In the high-temperature stage, the relative peak areas of Ar-H, COOH, and C=O gradually increased, while those of Ar-CH_3_ and CH_2_ decreased; the relative peak areas of C, CH, Ar-C, and Ar-O first increased and then decreased; while the bridging aromatic carbon first decreased and then increased.

In summary, the evolution of the molecular carbon structure in coal exhibits a phased pattern: at low temperatures, physical desorption and weak bond vibrations predominate; at medium temperatures, the breakdown of aliphatic side chains and the condensation of aromatic nuclei are central; and at high temperatures, the deep condensation of aromatic nuclei and the formation of aromatized structures dominate.

#### 2.4.2. Characteristics of the Evolution of Functional Group Structures

**Figure 12:**
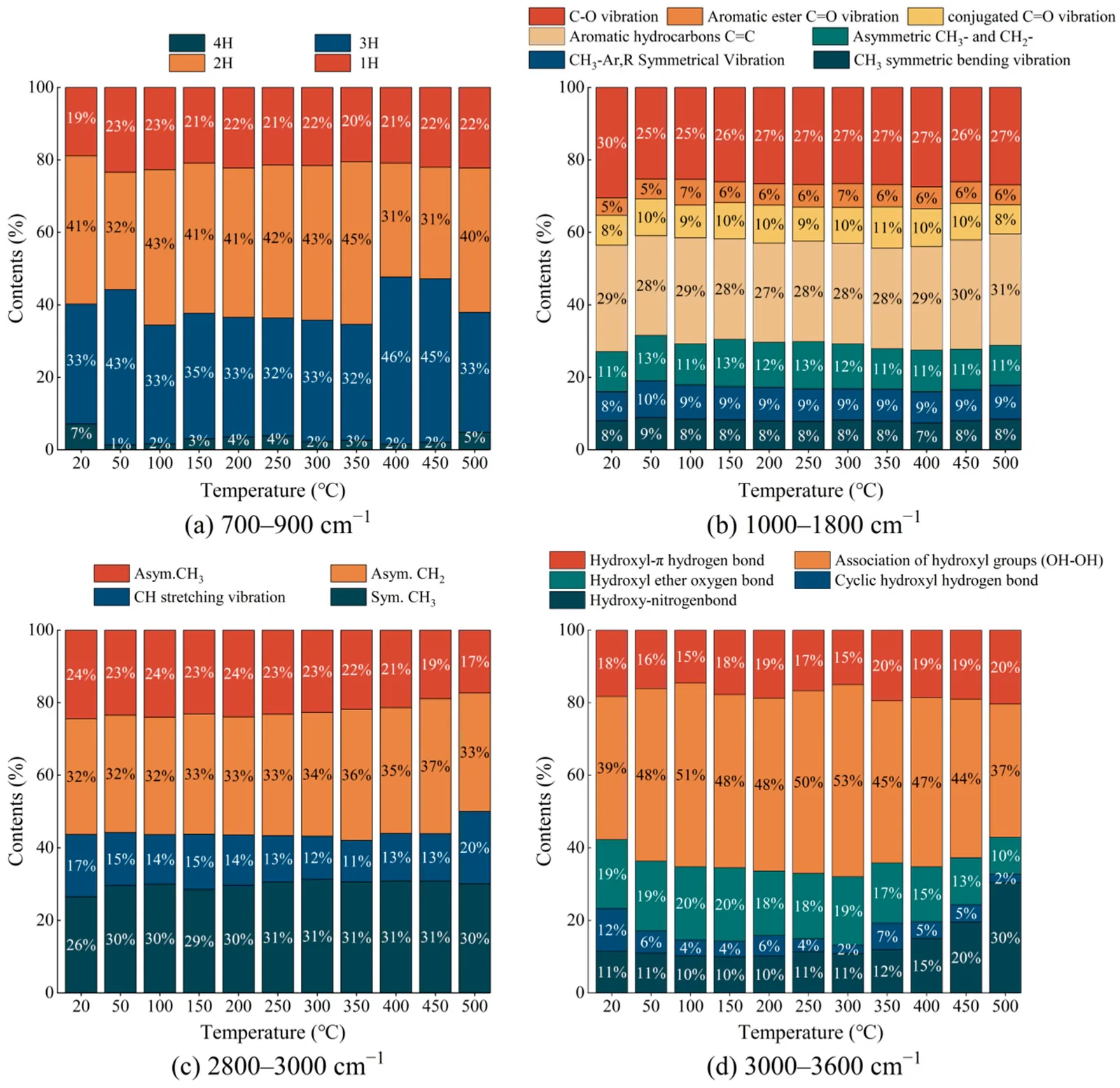
Changes in functional groups of coal samples at different temperatures: (**a**) 700–900 cm^−1^; (**b**) 1000–1800 cm^−1^; (**c**) 2800–3000 cm^−1^; (**d**) 3000–3600 cm^−1^.

FTIR analysis was performed on raw coal and samples heat-treated at 50–500 °C. The spectra were divided into four spectral regions based on characteristic absorption bands, and the changes in the content assigned to various functional groups are shown in Figure 12.

During the low-temperature phase, the molecular structure of coal remained stable, and changes in functional groups were minimal. In the 700–900 cm^−1^ region, the bands assigned to tri- and tetrasubstituted aromatic rings together accounted for approximately 75% of the total peak area, while those assigned to di-substituted aromatic rings had the lowest proportion, at 1.3%. In the 1000–1800 cm^−1^ region, the relative peak area of C-O vibrations of phenols, alcohols, ethers, and esters first decreased and then increased, while the asymmetric stretching vibration of CH_3_- and CH_2_- exhibited an “M”-shaped pattern; the remaining oxygen-containing functional groups showed minimal fluctuations. In the 2800–3000 cm^−1^ region, CH_2_ and CH_3_ stretching vibrations dominate, accounting for more than 80% of the content. The relative contribution of CH stretching vibrations decreased overall, while CH_2_ stretching increased. In the 3000–3600 cm^−1^ region, the OH-OH hydrogen bonds from self-associating hydroxyl groups accounted for the highest proportion, rising from 39.4% to 50.6% before falling to 47.6%. The relative peak area assigned to hydroxyl–ether hydrogen bonds (OH–O) followed a similar trend, while the hydrogen bonds from cyclic hydroxyl groups and hydroxyl-π bonds showed the opposite trend.

During the medium-temperature stage, the reactions are primarily characterized by the cleavage of fatty acid side chains and the rearrangement of hydroxyl groups. In the 700–900 cm^−1^ region, the relative peak areas associated with the four aromatic substitution patterns remained comparatively stable. In the 1000–1800 cm^−1^ region, the main contributing peaks were assigned to C-O vibrations of phenols, alcohols, ethers, and esters, and to aromatic C=C stretching, while the aromatic ester C=O band remained a minor component. In the 2800–3000 cm^−1^ region, as the temperature increased, the relative contribution of CH_2_ stretching vibration bands increased, while that of CH_3_ and CH stretching vibrations decreased. In the 3000–3600 cm^−1^ region, the contributions from self-associative hydroxyl groups (OH-OH) and hydroxyl-ether hydrogen bonds (OH-O) gradually increase, while those from cyclic hydroxyl hydrogen bonds and hydroxyl-π hydrogen bonds gradually decreased. These changes suggest a redistribution of hydroxyl hydrogen-bonding environments during this stage.

During the high-temperature phase, as the temperature rose, the relative peak area assigned to tetrasubstituted aromatic rings in the 700–900 cm^−1^ region initially decreased and subsequently increased, whereas that assigned to trisubstituted aromatic rings showed the opposite trend. These changes suggest variations in aromatic C-H substitution patterns. In the 1000–1800 cm^−1^ region, the relative peak area of aromatic C=C stretching bands showed an overall upward trend; the relative peak area of the conjugated C=O vibration band reached its minimum of 8.1% at 500 °C, while the other oxygen-containing functional groups exhibited slight fluctuations. In the 2800–3000 cm^−1^ region, the CH_3_ stretching vibration decreased, the CH_2_ asymmetric stretching vibration exhibited an “M”-shaped pattern, and the CH stretching vibration showed an overall upward trend, reaching a peak of 19.9% at 500 °C. In the 3000–3600 cm^−1^ region, the relative peak area of self-associating hydroxyl groups (OH-OH) decreased from approximately 52.9% to approximately 36.7%, but this component remained dominant at 500 °C. The trends in the aromatic C=C and CH_3_ bands are consistent with the ^13^C NMR results, which show increased Ar–H and decreased methyl carbon relative peak areas. The consistency between the trends in the aromatic and methyl-related spectral bands indicates a relative enrichment of aromatic components in the coal following high-temperature treatment.

In summary, the evolution of functional groups in coal occurs in distinct stages: at low temperatures, physical desorption and weak-bond vibrations predominate; at medium temperatures, the breakdown of aliphatic side chains and the restructuring of the hydroxyl network are central; and at high temperatures, the coal becomes relatively enriched in aromatic structures. These spectroscopic observations provide an experimental basis for discussing changes in functional-group distributions during heat treatment.

#### 2.4.3. Evolutionary Characteristics of Elemental Occurrence Forms

**Figure 13:**
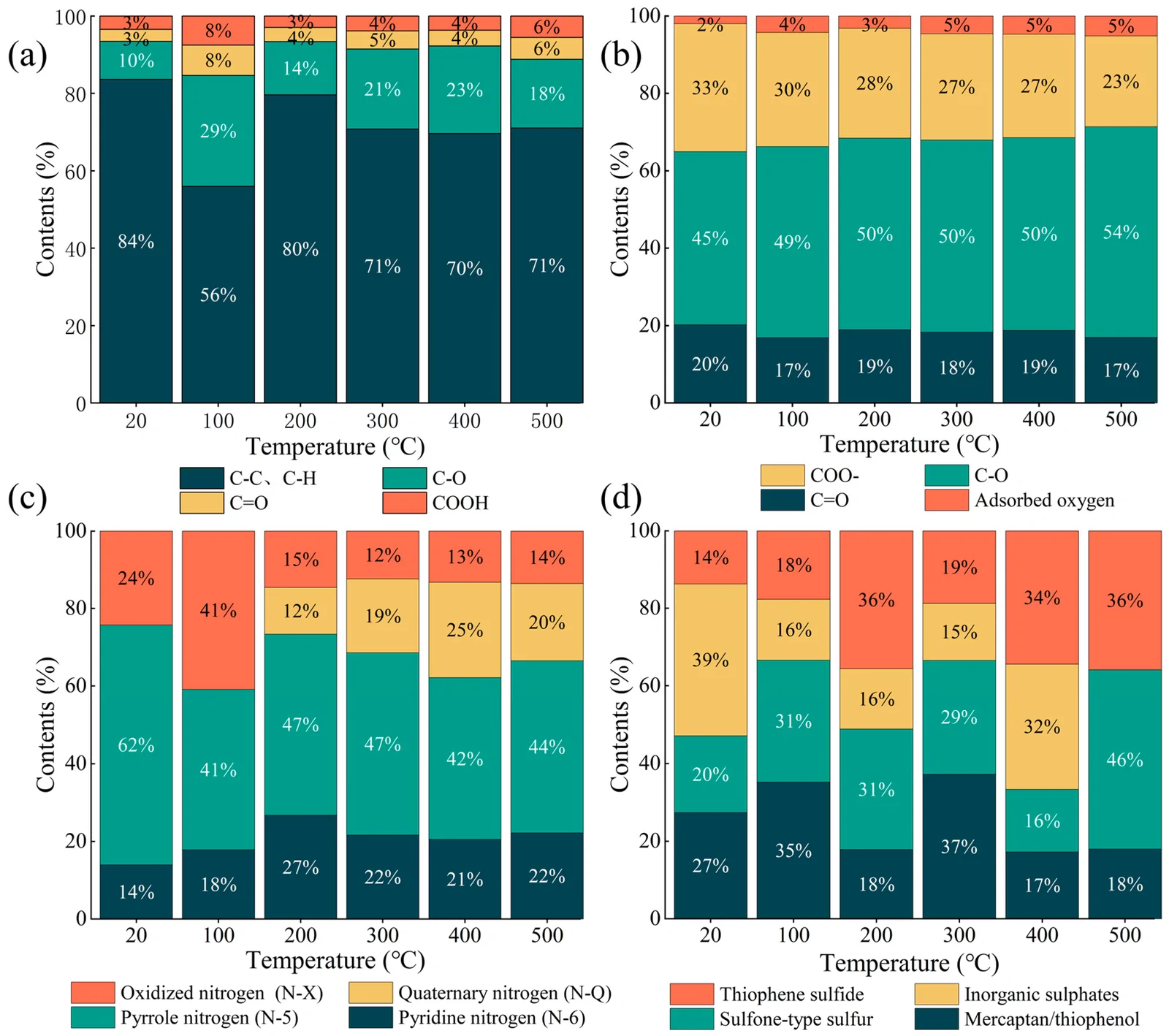
Changes in the contents of major elements by occurrence form: (**a**) C 1s; (**b**) O 1s; (**c**) N 1s; (**d**) S 2p.

The relative XPS peak areas were compared to characterize changes in the surface chemical states of C, O, N, and S, as shown in Figure 13.

In the low-temperature stage, functional groups are mainly affected by physical processes, and the chemical structure remains stable. Carbon in the coal sample exists predominantly as C-C and C-H bonds. As the temperature rises, the C-C and C-H bonds break, producing small amounts of C-O, C=O, and COOH. Oxygen in the coal predominantly exists in the form of C-O, while COO- participates in reactions to form C-O, and its relative peak area increased from 45% to 50%; N in coal primarily exists in the N-5 form; N-5 and N-X participate in reactions to form small amounts of N-Q and N-6; S in coal primarily participates in reactions in the form of inorganic sulfates, thiols, and thiophenes, yielding sulfone-type sulfur and thiophene sulfur.

During the medium-temperature stage, chemical bond cleavage and functional group rearrangement predominate. The relative peak areas of C-C and C-H in coal decreased, while the C-O content increased. C-C and C-H participate in reactions to form C-O, with their combined content decreasing from 80% to 71%. This change indicates an increased relative contribution of oxygenated carbon on the coal surface. Primarily N-6 and N-X in coal participate in reactions to form N-Q, and their relative peak area increased from 12% to 19%. The thiophene sulfur primarily reacts to form mercaptan/thiophenol.

The high-temperature stage is characterized by deep condensation of aromatic nuclei and the stabilization of heteroatom functional groups. The forms in which carbon is present in the coal remain relatively stable; the C-O content first increased and then decreased, rising from 21% to 23% before falling to 18%. In the O 1s spectrum, the relative peak area of C-O increased overall, while that of COO- decreased. Among nitrogen species, N-5 remains the predominant component, with N-X exhibiting a continuous upward trend, while N-Q initially increased and then decreased. During the reaction process, inorganic sulfates, thiols, and phenols participate in reactions to form sulfone sulfur and thiophene sulfur.

In summary, the evolution of C, O, N, and S functional groups in coal occurs in distinct stages: at low temperatures, the process is dominated by physical desorption and functional group rearrangement; at medium temperatures, it centers on chemical bond cleavage and the transformation of heteroatom functional groups; and at high temperatures, it is characterized by aromatic condensation and the stabilization of heteroatom functional groups. The relative areas of the C, O, N, and S chemical components all adjusted with the temperature process from 20 to 500 °C. Among these, the variations in C 1s and O 1s were relatively small between 300 and 500 °C, while the composition ratios of N 1s and S 2p continued to exhibit significant fluctuations.

#### 2.4.4. Evolutionary Characteristics of Aromatic Stripe Structure

**Figure 14:**
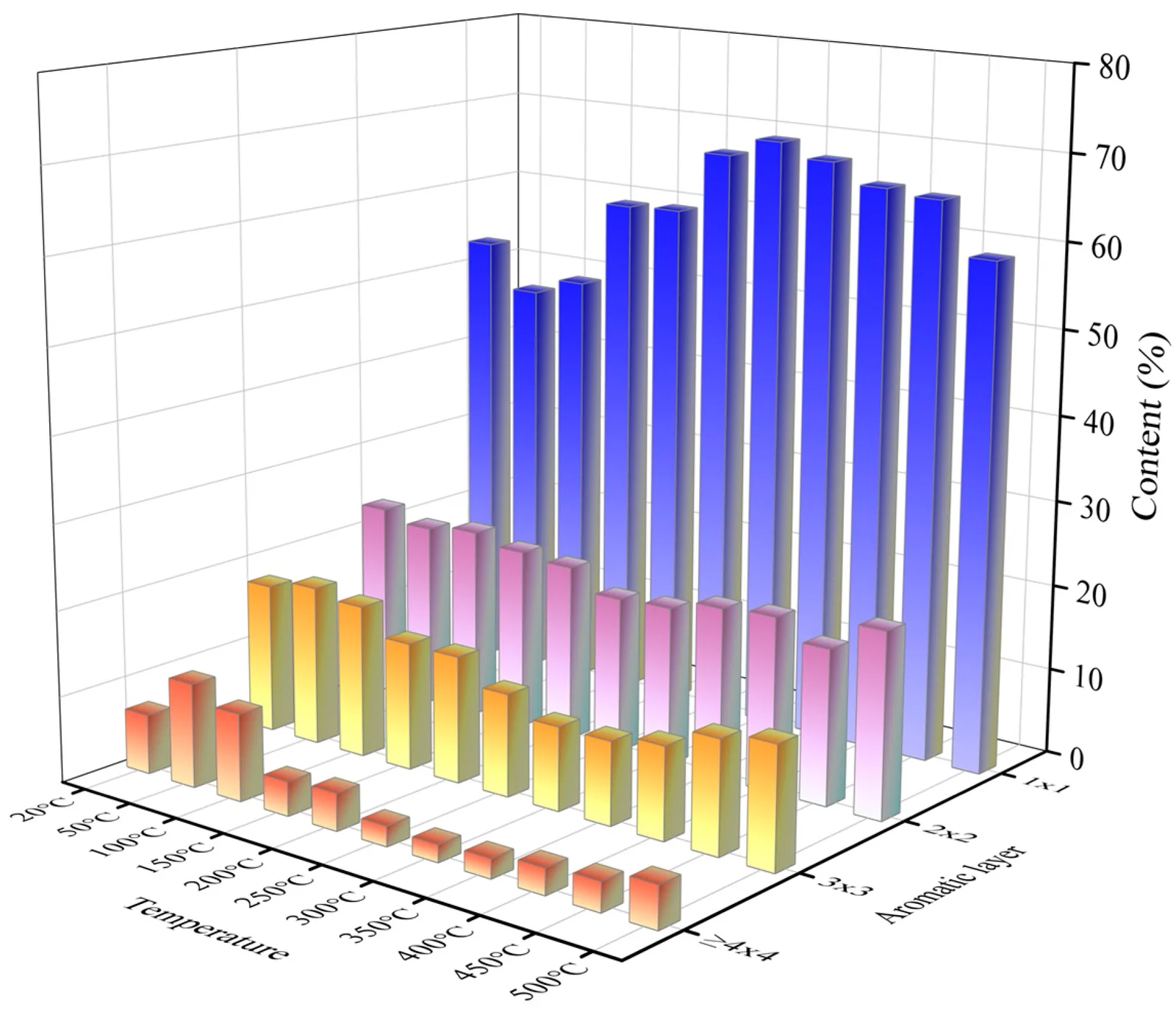
Changes in the length distribution of aromatic stripes at different temperatures.

HRTEM images were acquired at 11 temperature points, with one image collected at each point. The images were processed using DigitalMicrograph 3.6 and Python 3.12.0, with approximately 1000 valid aromatic fringes counted per image. The resulting aromatic lattice fringe length histograms are shown in Figure 14 and each histogram represents the distribution within a single field of view at the corresponding temperature. At all temperatures, the aromatic layers in the samples were predominantly 1 × 1 aromatic layers, followed by 2 × 2 and 3 × 3 aromatic layers, while the proportion of individual 4 × 4 and larger aromatic layers was relatively low. This distribution pattern is consistent with the results of previous studies [7,26].

As the heat treatment temperature increased, the aromatic fringe-length distributions exhibited temperature-dependent changes. During the low-temperature stage, oxygen-containing functional groups and bridging bonds break; the proportion of the 1 × 1 aromatic layers first decreased and then increased, reaching a minimum of 47.6% at 50 °C. The proportion of 2 × 2 aromatic layers shows an overall downward trend, while the proportions of 3 × 3 and ≥4 × 4 aromatic layers first increased and then decreased, indicating that an initial rearrangement of the coal structure occurs during this stage. During the medium-temperature stage, the proportion of 1 × 1 aromatic layers increased from 60% to 69.6%, whereas other aromatic layers decreased. These changes indicate that thermal treatment promoted the cracking of larger aromatic layers and the formation of short stripes. Upon entering the high-temperature phase, the relative proportion of the 1 × 1 gradually decreased, accompanied by an overall increased in longer fringes. This reflects further polycondensation and aromatization of smaller aromatic structures, leading to the gradual formation of larger aromatic layers. In contrast, the 2 × 2 aromatic layers exhibited only minor fluctuations throughout the entire heating process, indicating that they are relatively stable transitional aromatic structural units.

### 2.5. The Relationship Between Molecular Structure Evolution and Thermal Reactivity

During the low-temperature stage, the carbon skeleton of the coal sample consists primarily of aromatic carbon, and the functional groups and aromatic striping patterns vary with heat treatment temperature. In the range of 20–200 °C, aromatic carbon constitutes the main component of the coal sample’s carbon skeleton, while aliphatic carbon consists primarily of methyl and methylene carbon. The proportions of C-O, C=O, and COO-/COOH in the O 1s spectrum changed, while 1 × 1 aromatic layers consistently dominated the HRTEM images. Compared to raw coal, the onset temperature of pyrolysis (T_2_) for low-temperature heat-treated coal samples showed limited change, while the ignition temperature (T_3_), maximum combustion rate temperature (T_4_), and complete burnout temperature (T_5_) generally shifted toward higher temperatures.

During the medium-temperature stage, structural changes in the coal samples were primarily manifested as the rearrangement of C-O and aromatic carbon types. In the 200–300 °C range, the relative peak area of C-O in the C 1s spectrum increased, the content of bridging aromatic carbon (C-C) bonds in the aromatic carbon increased, and in the aliphatic structure, the content of R-CH_3_ decreased while that of -CH_2_ increased, leading to a further rise in the proportion of 1 × 1 aromatic layers. During this stage, changes occurred in the carbon structural composition and the distribution of aromatic layers, but T_2_ and T_3_ showed only slight fluctuations.

During the high-temperature stage, the aromatic structure in the coal sample became relatively enriched, and T_2_ and T_3_ shifted overall toward higher temperatures. In the 300–500 °C range, the relative peak area of Ar-H and aromatic C=C stretching vibrations in the aromatic carbon increased, while the relative peak area of -CH_2_, R-CH_3_, and Ar-CH_3_ in the aliphatic fraction decreased overall, and the content of CH_3_ stretching vibrations in the FTIR spectrum decreased. Both the ^13^C-NMR and FTIR results indicate a decrease in aliphatic components and a relative enrichment of aromatic structures. The proportion of 3 × 3 and ≥4 × 4 aromatic fringes increased overall, providing scale information to complement the changes in chemical composition. During this stage, T_4_ and T_5_ showed little variation among the heat-treated coal samples, while the relative enrichment of aromatic structures corresponded with the increase in T_2_ and T_3_.

Overall, the structural changes observed across the heat-treatment series are consistent with the stage-dependent thermal reaction characteristics. The low-temperature stage is characterized primarily by changes in the distribution of functional groups and aromatic layers, with aromatic carbon consistently dominating; the medium-temperature stage is characterized by a relative enrichment of C-O and bridging aromatic carbon (C-C) bonds; and the high-temperature stage is characterized by a decrease in aliphatic carbon content and a relative enrichment of aromatic structures. The T_3_, T_4_, and T_5_ values of the thermally treated coal samples were generally higher than those of the raw coal, and T_2_ and T_3_ values increased further during the high-temperature stage. Multispectral characterization and thermal analysis results collectively indicate that the observed changes in functional group composition, carbon skeleton structure, and aromatic fringe length distribution are consistent with the stage-dependent macroscopic thermal reaction characteristics, providing an experimental basis for discussing the structural changes accompanying the thermal responses of Kerke coal.

## 3. Materials and Methods

### 3.1. Coal Samples and Heat Treatment

To investigate the structural evolution characteristics of raw coal during the heat treatment process at the Kerke Mining Area in Yili, Xinjiang, this study selected raw coal from this mining area as the subject of research. The coal was crushed and sieved, and the fraction passing through a standard 200-mesh sieve was collected. In an SRJX-4-13 muffle furnace (Beijing Yongguangming Medical Instrument Factory, Beijing, China), heat treatment was performed by heating the sample in static air at a rate of 10 °C min^−1^ to temperatures of 50, 100, 150, 200, 250, 300, 350, 400, 450, and 500 °C. After the furnace reached each target temperature, the samples were held at that temperature for 1 h, then removed and allowed to cool to room temperature. A total of 10 groups of heat-treated coal samples were prepared.

### 3.2. Proximate Analysis and Ultimate Analysis

The Proximate analysis was conducted according to GB/T 212-2008 [33]. Elemental analysis was performed in accordance with GB/T 476-200 [34] using a UNICUBE elemental analyzer (Elementar Analysensysteme GmbH, Langenselbold, Germany). The atomic ratios were calculated through normalization.

### 3.3. Spectral Characterization

#### 3.3.1. ^13^C-NMR

For the ^13^C-NMR measurements, spectra were acquired using a fully digital, superconducting AVANCE III 600 MHz nuclear magnetic resonance spectrometer (Bruker BioSpin AG, Fällanden, Switzerland). The instrument was equipped with a dual-resonance MAS probe, a 4.0 mm high-resolution rotor, a rotor speed of 10 kHz, a pulse width of 4 μs, a pulse delay time of 1 s, a contact time of 2 ms, and a total of 10,000 scans.

#### 3.3.2. FTIR

FTIR testing was conducted using a VERTEX 70 Fourier-transform infrared spectrometer (Bruker Optik GmbH, Ettlingen, Germany) to acquire infrared spectra. The spectral scan range was 4000–400 cm^−1^, with a resolution of 4 cm^−1^, and a total of 120 scans were performed.

#### 3.3.3. XPS

XPS testing was performed using an ESCALAB 250Xi X-ray photoelectron spectrometer (Thermo Fisher Scientific, East Grinstead, UK), in accordance with GB/T 34326-2017 [35]. Monochromatic Al Kα X-ray was used as the excitation source, with the power set to 200 W. The binding energy was calibrated using the C 1s peak at 284.8 eV as a reference.

### 3.4. HRTEM

HRTEM characterization was performed using a JEM-2100 high-resolution transmission electron microscope (JEOL Ltd., Akishima, Japan). The accelerating voltage was set to 200 kV, with a point resolution of 0.23 nm and a line resolution of 0.14 nm. The magnification reached 1 × 106 in high-magnification mode and 6 × 104 in low-magnification mode.

### 3.5. TG

An STA7300 simultaneous thermal analyzer (Hitachi High-Tech Science Corporation, Tokyo, Japan) was used to programmatically heat the sample from 30 °C to 1200 °C at a rate of 10 °C/min in an air atmosphere, with a gas flow rate of 200 mL/min.

### 3.6. Kinetic Calculations

The apparent activation energy of coal was calculated using the Coats–Redfern integral method. A first-order reaction model was adopted for the selected reaction stages, with the equations given below:

(1) $$g\left(\alpha \right)=\int_{0}^{\alpha }\frac{\mathrm{du}}{1-u}=-\ln\left(1-\alpha \right)$$

Here, α denotes the conversion, calculated from the mass change as follows:

(2) $$\alpha =\frac{m_{0}-m_{T}}{m_{0}-m_{f}}$$

where m_T_ is the sample mass at temperature, and m_0_ and m_f_ are the initial and final reference masses used to calculate conversion, respectively.

Under the approximation $\frac{2\mathrm{RT}}{E_{a}}\leq 1$, the Coats–Redfern equation can be expressed as

(3) $$\ln\left[\frac{-\ln\left(1-\alpha \right)}{T^{2}}\right]\approx \ln\left(\frac{\mathrm{AR}}{\beta E_{a}}\right)-\frac{E_{a}}{\mathrm{RT}}$$

A is the pre-exponential factor (min^−1^), β is the heating rate (K min^−1^), E_a_ is the apparent activation energy (kJ mol^−1^), R is the gas constant (8.314 J mol^−1^ K^−1^), and T is the absolute temperature (K).

For each stage, the apparent activation energy was determined by linear regression of $\ln\left[\frac{-\ln\left(1-\alpha \right)}{T^{2}}\right]$ against $\frac{1}{T}$. All fits were performed over the same conversion range from 0.1 to 0.9, and the corresponding R^2^ values are shown in Table 10. Because the analysis uses a single heating rate and an assumed reaction model, the values should be interpreted as model-dependent apparent activation energies under the tested conditions.

## 4. Conclusions

By integrating elemental composition and structural parameters obtained from various characterization methods, the molecular formula of the Yili Kerke coal was determined to be C_179_H_132_N_2_O_13_. Structural optimization was performed using a combination of MM and MD simulations, yielding the lowest-energy conformation with a total model energy of 661.38 kcal mol^−1^. The calculated density was 1.31 g cm^−3^. The simulated spectrum of the molecular structure model showed good agreement with the experimentally measured ^13^C-NMR spectrum. Future comparison of calculated and experimental FTIR spectra could provide a complementary assessment of the model’s functional-group representation.

Macroscopic thermal reaction results show that the ignition temperature (T_3_), maximum combustion rate temperature (T_4_), and complete burnout temperature (T_5_) of the heat-treated coal samples were all higher than those of the raw coal. The pyrolysis onset temperature (T_2_) and T_3_ generally increased after high-temperature treatment. The apparent activation energy in Stage IV was higher than in other stages and reached its maximum in coal samples treated at 500 °C; the apparent activation energy in Stage III fluctuated significantly.

Results on microstructural evolution show that during the low-temperature stage, aromatic carbon consistently dominated, while the relative proportions of oxygen-containing functional groups such as C-O changed; during the medium-temperature stage, C-O and aromatic bridgehead carbon were relatively enriched, the relative peak area of R-CH_3_ decreased, and that of CH_2_ increased; during the high-temperature stage, the relative peak area of Ar-H increased from 37% to 50%, while that of -CH_2_ decreased from 11% to 3%; the relative peak area of aromatic hydrocarbons C=C increased, indicating a relative decrease in aliphatic carbon and a relative enrichment of aromatic structures.

The observed structural changes are consistent with the stage-dependent thermal responses of the heat-treated coal samples. The low-temperature stage is characterized primarily by changes in functional groups and the distribution of aromatic layers, with aromatic carbon consistently dominating and T_3_, T_4_, and T_5_ increasing; changes in structural composition and layer distribution during the medium-temperature stage correspond to slight fluctuations in T_2_ and T_3_; in the high-temperature stage, the relative enrichment of aromatic structures corresponds to a shift in T_2_ and T_3_ toward higher values, while T_4_ and T_5_ show little variation among heat-treated coal samples, providing a structural basis for explaining the differences in the thermal response of Kerke coal.

## Figures and Tables

**Table 1:** Proximate and ultimate analysis of the Kerke coal.

Proximate Analysis (%)				Ultimate Analysis (%)					Atomic Ratio			
M_ad_	A_ad_	V_ad_	FC_ad_	C_daf_	H_daf_	O_daf_	N_daf_	S_daf_	H/C	O/C	N/C	S/C
1.57	2.15	32.13	64.15	84.82	5.25	8.27	1.42	0.25	0.74	0.07	0.01	0

**Table 2:** Structural parameters derived from peak fitting of ^13^C-NMR spectra of coal samples.

	Peak Type	Peak Center	Peak Area	Relative Peak (%) Area%	Functional Group
Aliphatic carbon 33.66%	Gaussian	12.74	230.89	3.27	R-CH_3_
	Gaussian	20.19	543.93	7.70	Ar-CH_3_
	Gaussian	30.23	890.59	12.61	-CH_2_
	Gaussian	39.53	423.08	5.99	-C-, -CH
	Gaussian	50.24	288.77	4.09	O-CH_3_, O-CH_2_
Aromatic carbon 66.34%	Gaussian	108.30	282.12	4.00	Ar-H
	Gaussian	119.99	1130.44	16.01	Ar-H
	Gaussian	127.63	1476.36	20.91	Ar-H
	Gaussian	136.80	700.00	9.91	Aromatic bridgehead carbon
	Gaussian	143.01	415.85	5.89	Ar-C
	Gaussian	153.43	486.90	6.90	Ar-O
	Gaussian	168.26	108.80	1.54	-COOH
	Gaussian	198.46	83.89	1.19	-C=O

**Table 3:** Structural parameters of coal samples.

f_a_	f_a_^C^	f_a_′	f_a_^N^	f_a_^H^	f_a_^P^	f_a_^S^	f_a_^B^	f_al_	f_al_^*^	f_al_^H^	f_al_^O^
66.34	2.73	63.61	22.70	40.91	6.90	5.89	9.91	33.66	10.97	18.60	4.09

Note: f_a,_ total aromatic carbon; f_a_^C^, carbonyl and carboxyl carbon; f_a_**′**, aromatic carbon; f_a_^N^, nonprotonated aromatic carbon; f_a_^H^, protonated aromatic carbon; f_a_^P^, hydroxyl or etheroxyl aromatic carbon; f_a_^S^, alkylated aromatic carbon; f_a_^B^, aromatic bridgehead carbon; f_al_, the total aliphatic carbon; f_al_^*^, methyl carbon or quaternary carbon; f_al_^H^, methylene or methine carbon; f_al_^O^, oxygenated aliphatic carbon.

**Table 4:** Infrared spectral fitting and peak assignment of coal samples.

	Number	Peak Type	Peak Position (cm^−1^)	Peak Area	Relative Peak Area (%)	Assignment
Aromatic Structures	1	Gaussian	740.07	1.04	7.10	Benzene ring disubstitution (4H)
	2	Gaussian	750.75	1.55	10.60	Benzene ring trisubstitution (3H)
	3	Gaussian	766.54	1.81	12.32	Benzene ring trisubstitution (3H)
	4	Gaussian	791.01	1.49	10.20	Benzene ring trisubstitution (3H)
	5	Gaussian	811.07	2.39	16.30	Tetrasubstitution of benzene ring (2H)
	6	Gaussian	827.62	1.92	13.08	Tetrasubstitution of benzene ring (2H)
	7	Gaussian	847.85	1.69	11.51	Tetrasubstitution of benzene ring (2H)
	8	Gaussian	868.30	2.02	13.76	Benzene ring pentasubstitution (1H)
	9	Gaussian	883.62	0.75	5.13	Benzene ring pentasubstitution (1H)
Oxygen-Containing Functional Groups	1	Gaussian	1037.49	1.77	0.75	C-O vibrations in phenols, alcohols, ethers, and esters
	2	Gaussian	1088.09	6.03	2.57	C-O vibrations in phenols, alcohols, ethers, and esters
	3	Gaussian	1145.00	8.42	3.60	C-O vibrations in phenols, alcohols, ethers, and esters
	4	Gaussian	1187.44	16.27	6.94	C-O vibrations in phenols, alcohols, ethers, and esters
	5	Gaussian	1288.36	19.22	8.20	C-O vibrations in phenols, alcohols, ethers, and esters
	6	Gaussian	1243.20	19.67	8.39	C-O vibrations in phenols, alcohols, ethers, and esters
	7	Gaussian	1340.26	18.70	7.98	CH_3_ symmetric bending vibration
	8	Gaussian	1393.21	18.94	8.08	CH_3_-Ar,R symmetrical vibration
	9	Gaussian	1448.85	25.92	11.06	Asymmetric CH_3_- and CH_2_-
	10	Gaussian	1612.86	33.70	14.38	Aromatic hydrocarbons C=C
	11	Gaussian	1500.11	18.79	8.02	Aromatic hydrocarbons C=C
	12	Gaussian	1566.72	16.24	6.93	Aromatic hydrocarbons C=C
	13	Gaussian	1665.24	19.29	8.23	(π-π) conjugated C=O vibration
	14	Gaussian	1726.25	11.37	4.85	Aromatic ester C=O vibration
Aliphatic hydrocarbons	1	Gaussian	2857.62	7.50	26.49	CH_3_ symmetric stretching vibration (Sym.CH_3_)
	2	Gaussian	2895.90	4.88	17.22	CH stretching vibration
	3	Gaussian	2924.40	9.01	31.81	CH_2_ asymmetric stretching vibration (Asym.CH_2_)
	4	Gaussian	2956.72	6.93	24.48	CH_3_ asymmetric stretching vibration (Asym.CH_3_)
hydroxyl group	1	Gaussian	3049.21	4.58	11.47	OH-N hydrogen bond
	2	Gaussian	3200.00	4.70	11.77	Cyclic hydroxyl hydrogen bond
	3	Gaussian	3291.94	7.58	19.01	Hydroxyl ether hydrogen bond
	4	Gaussian	3382.62	10.19	25.56	OH-O hydrogen bond
	5	Gaussian	3456.47	5.53	13.87	Association of hydroxyl groups (OH-OH)
	6	Gaussian	3535.37	7.30	18.30	OH-π hydrogen bond

**Table 5:** Elemental forms and relative abundances in XPS.

Element	Group	Binding Energy E/eV	Relative Peak (%) Abundance/%
C 1s	C-C, C-H	284.8	83.62
	C-O	286.35	9.84
	C=O	287.72	3.11
	O-C=O	289.27	3.43
O 1s	C=O	531.51	20.22
	C-O	532.5	44.75
	COO-/COOH	533.7	33.02
	Adsorbed oxygen	536.25	2.00
N 1s	Pyridinic nitrogen (N-6)	398.56	13.95
	Pyrrolic nitrogen (N-5)	400.42	61.78
	Oxidized nitrogen (N-X)	402.83	9.85
	Oxidized nitrogen (N-X)	406.01	14.42
S 2p	Inorganic sulphates	169.97	39.26
	Sulfone-type sulfur	168.77	19.68
	Mercaptan/thiophenol	163.98	27.37
	thiophenic sulfur	165.18	13.69

**Table 6:** Types and quantities of aromatic structures in coal.

Type	Quantity	Type	Quantity
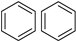	3	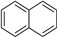	5
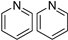	1	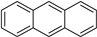	2
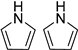	1	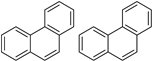	1

**Table 7:** Mass fraction of elements in the Kerke coal structural model.

Molecular Formula	Molecular Weight	Elemental Mass Fraction (%)					Experimental Element Fraction (%)				
		C	H	O	N	S	C_daf_	H_daf_	O_daf_	N_daf_	S_daf_
C_179_H_132_O_13_N_2_	2516	85.37	5.25	8.27	1.11	0	84.82	5.25	8.27	1.42	0.25

**Table 8:** Energy changes during the coal molecule optimization process.

Status	Total Energy (kcal mol^−1^)	Electronic Energy (kcal mol^−1^)				Non-Bonding Energy (kcal mol^−1^)		
		E_B_	E_A_	E_T_	E_I_	E_H_	E_van_	E_E_
Initial	399,527.36	1887.75	1773.74	1156.14	73.89	0	394,656.31	−20.46
Geometric Optimization	799.48	99.76	186.54	198.5	2.75	0	345.34	−33.41
Annealing Simulation	661.38	89.39	134.63	202.75	4.29	0	272.8	−42.48

Notes: E_B_ = E_Bond_; E_A_ = E_Angle_; E_T_ = E_Torsion_; E_H_ = E_Hydrogen bond_; E_van_ = E_van der Waals_; E_I_ = E_inversion_; E_E_ = E_electrostatic_.

**Table 9:** Characteristic temperatures of coal samples at different temperatures.

Temperature (°C)	T_1_	T_2_	T_3_	T_4_	T_5_
20	199.43	313.98	445.41	518.69	606.26
50	201.36	310.56	478.09	558.42	625.73
100	190.82	312.10	457.99	558.19	622.28
150	234.51	307.24	473.64	553.97	618.55
200	223.03	304.50	468.52	554.99	621.44
250	215.42	315.46	477.44	552.58	617.13
300	183.71	317.72	468.86	556.27	622.54
350	228.98	313.43	477.45	552.74	623.97
400	220.40	324.94	472.67	560.94	628.62
450	187.34	343.23	492.46	562.89	625.58
500	194.91	344.60	502.64	554.33	620.46

**Table 10:** R^2^ of the linear fits for the five stages.

Temperature (°C)	Stage I	Stage II	Stage III	Stage IV	Stage V
20	0.981	0.9792	0.9969	0.9963	0.9876
50	0.9832	0.9968	0.9438	0.9652	0.9789
100	0.9795	0.9908	0.9902	0.9641	0.9804
150	0.984	0.9969	0.9336	0.9671	0.9523
200	0.9838	0.9975	0.9558	0.9704	0.9659
250	0.9819	0.9966	0.9641	0.9836	0.9921
300	0.9987	0.9519	0.9939	0.9868	0.9847
350	0.9841	0.9979	0.9314	0.9842	0.9528
400	0.9835	0.9964	0.9211	0.9788	0.9866
450	0.9608	0.9846	0.9946	0.9845	0.9875
500	0.9823	0.9922	0.9676	0.9987	0.9903

## Data Availability

The results of this study are reported in the article. Access to some supporting data is currently restricted because these data are being used in ongoing research. Requests for access should be addressed to the corresponding author.

## Abbreviations

The following abbreviations are used in this manuscript:

TG	Thermogravimetric analysis
FTIR	Fourier transform infrared spectroscopy
XPS	X-ray photoelectron spectroscopy
^13^C-NMR	Solid-state 13C nuclear magnetic resonance spectroscopy
HRTEM	High resolution transmission electron microscopy
MM	Molecular mechanics
MD	Molecular dynamics
